# Cheminformatics-based screening and evaluation of phytochemicals as CDK2 inhibitors in colorectal cancer therapy

**DOI:** 10.1371/journal.pone.0331438

**Published:** 2025-09-03

**Authors:** Nowshin Tabassum, Md. Ekram Hossan, Md. Mujahidul Islam, Mahmudul Hasan, Md Salamoon Islam, Sunjida Masud, Firoz Ahmed, Noimul Hasan Siddiquee

**Affiliations:** 1 Bioinformatics Laboratory (BioLab), Noakhali, Bangladesh; 2 Department of Microbiology, Noakhali Science and Technology University, Noakhali, Bangladesh; 3 Department of Biochemistry and Molecular Biology, University of Chittagong, Chittagong, Bangladesh; 4 Department of Chemistry, Jashore University of Science and Technology, Jashore, Bangladesh; University of Mashreq, IRAQ

## Abstract

Colorectal cancer (CRC) poses a significant global health issue. It ranks as the third most common type of cancer and the second leading cause of cancer-related deaths. Among the molecular factors driving its progression, cyclin-dependent kinase 2 (CDK2) plays a key role. CDK2 is a protein kinase essential for regulating the cell cycle, and its dysregulation is implicated in the development of various cancers, notably CRC. Fruquintinib is an already available drug against CRC. However, this study is being performed in search of better drug-like compounds. Some studies have shown that phytochemicals are less toxic and have fewer adverse effects than commercially available medications. With the vision of detecting CDK2 inhibitors, phytochemicals with anticancer activity can be used as alternatives to develop the drug candidate. Cheminformatics-based analysis is used for this purpose. This approach includes molecular docking, adsorption, distribution, metabolism, excretion/toxicity (ADME/T), post-docking molecular mechanism generalized born surface area (MM-GBSA), structural activity relationship (SAR), frontier molecular orbital (FMO), and molecular dynamics (MD) simulations. Molecular docking was employed to determine the binding strength of 4433 phytochemicals with anti-cancer properties sourced from the IMPPAT database. The top five candidates, CIDs-135438111, 6474893, 44257567, 10469828, and 353825, were selected based on their docking scores. Later, three lead compounds, CIDs-6474893, 10469828, and 135438111, were finalized depending on their favorable ADME/T profiles. All three selected pharmaceuticals demonstrated excellent post-dock MM-GBSA scores and HOMO-LUMO energy gaps, which served as confirmation of their efficacy and safety. The SAR analysis also revealed anti-mutagenic, antineoplastic, and apoptosis-inducing properties of the compounds. Finally, the rigidity of the protein-ligand complex structures was verified by MD simulations. Overall, the study suggests these three phytochemicals exhibit stronger binding and better pharmacological profiles than the control (fruquintinib), offering a promising direction for CRC treatment development.

## 1. Introduction

Cancer ranks as the second leading cause of death globally [1]. CRC stands as the fourth predominant malignancy and the third most significant contributor of cancer-related deaths worldwide [2]. CRC affects individuals of all ages, though a higher incidence is observed in those over 50 [3,4]. Contrary to popular belief, the lifetime average risk of colorectal cancer (CRC) is only 5% worldwide, and studies reveal that low and middle-income nations would see a 70% increase in CRC incidence by 2030 [2,5]. Abnormal mitosis and proliferation of colon cells are the causes of CRC [6], with genetic factors [7], lifestyle, habits, and diet being major contributors [8]. Risk factors encompass obesity, smoking, drinking alcohol, and diets rich in fat [9]. In contrast, genetic factors, like familial adenomatous polyposis coli (FAP) and hereditary non-polyposis colon cancer (HNPCC, Lynch syndrome), also exert considerable influence [10]. Although colorectal cancer may be asymptomatic in its initial phases, symptoms including rectal bleeding, decreased frequency of bowel movements, rectal bleeding, abdominal pain, weakness, and weight loss manifest as the disease progresses [11].

Colorectal cancer treatment has conventionally depended on chemotherapy, utilizing agents such as fluoropyrimidine and oxaliplatin, along with the surgical excision of tumors and metastases. In some instances, neoadjuvant or adjuvant chemotherapy or radiation may be administered before or after surgery to shrink and stabilize the tumor optimally [12]. However, existing therapeutic strategies are linked to significant side effects and are frequently worsened by drug resistance [13,14]. Consequently, the pursuit of novel, more precise treatments persists as researchers endeavour to create medicines that are more targeted and efficacious [15]. Recent years have witnessed significant advancements in molecular targeting, particularly targeting the vascular endothelial growth factor (VEGF), CDKs, and epidermal growth factor receptor (EGFR) [10].

The formation of new blood vessels associated with tumor growth is impeded by a VEGFR inhibitor known as fruquintinib [16]. Fruquintinib has been utilized to treat metastatic colorectal cancer (mCRC) by targeting the tyrosine kinase linked to VEGFR-1, VEGFR-2, and VEGFR-3, respectively [17]. Due to its strong suppression of VEGFR-1, 2, and 3 receptors, fruquintinib inhibits angiogenesis, which hinders tumor growth, and it inhibits lymphangiogenesis, which hinders tumor dispersion [18]. In vivo, oral fruquintinib treatment significantly reduced VEGF-induced VEGFR2 phosphorylation [19]. Through anti-angiogenesis, fruquintinib inhibited tumor growth in a patient-derived tumor xenograft model while preserving a favorable dose-response relationship [20]. Fruquintinib and anti-PD-1 (Programmed death receptor-1) therapy together may improve the antitumor immune response in microsatellite-stable colorectal cancer (MSS CRC) [21]. A monoclonal antibody that targets PD-1 is called sintilimab. When used in mice models with MC38 or CT26 xenograft tumors, which stand in for MSS and microsatellite-instability colorectal cancer, fruquintinib and sintilimab significantly inhibited tumor growth and extended survival time when compared to either drug alone [22].

In recent years, natural products like phytochemicals have gained attention for cancer therapy due to their distinctive structures, reduced toxicity, cost efficiency, especially in impoverished countries, and capacity to target several cancer-associated signaling pathways [23,24]. More than 60% of anticancer drugs originate from natural sources, and studies have shown that phytochemicals can disrupt cancer growth by altering pathways such as cellular proliferation, apoptosis, and angiogenesis [25,26]. *Withania somnifera*’s phytochemicals show promising anticancer properties with fewer side effects than synthetic drugs. Targeting cancer stem cells improves treatment outcomes and reduces recurrence [27]. The oxidative stress and inflammation that are associated with cancer growth are alleviated by their antioxidant and anti-inflammatory properties, which also influence cancer mechanisms such as apoptosis [27–29]. Natural chemicals such as terpenoids, alkaloids, and flavonoids present varied structures and modes of action for the identification of anticancer drugs. They can trigger apoptosis, impede cell proliferation, halt cell cycles, limit metastasis, and possess chemopreventive capabilities [30]. Additionally, they exhibit resistance mitigation, rendering them potent anticancer agents, and display various biological activities [31]. Phytochemicals have low toxicity, multi-target mechanisms, synergistic interactions [32], varied chemical structures, provide specific mechanisms [33], biological activity, diverse methods of action, bioavailability, accessibility, and synergistic effects, rendering them safer, more sustainable, and perhaps mitigating medication resistance [34]. The significance of phytochemicals in chemosensitization offers a possible approach for overcoming drug resistance, an essential barrier in colorectal cancer treatment [35].

**Fig 1:**
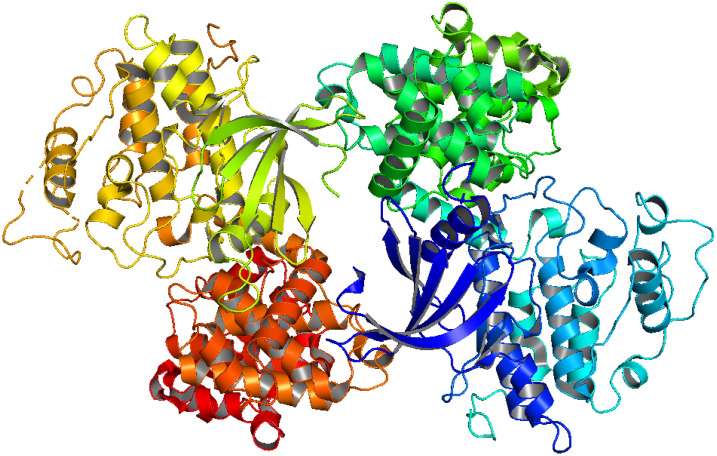
CDK2 protein (PDB ID-6GUE).

**Fig 2:**
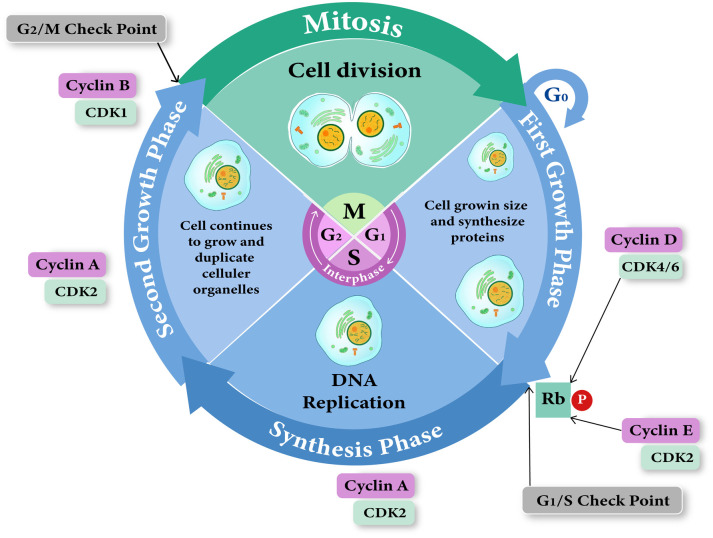
The CDK/cyclin complexes and the cell cycle phases.

CDK-2 (**Fig 1**) is essential in cell cycle regulation and is overexpressed in colorectal cancer, among other malignancies, rendering it an attractive target for drug development [36,37]. The phosphorylation of targets implicated in DNA replication is regulated by CDK2 in complex with cyclin A during the S phase of the cell cycle, as illustrated in **Fig 2** [38]. The Rb (retinoblastoma) protein is a protein that inhibits tumor development and is responsible for the regulation of the cell cycle, differentiation, and apoptosis [39]. Phosphorylation of the retinoblastoma (Rb) protein is a key factor in regulating the cell cycle [40]. The Rb protein has many phosphorylation sites that are targeted by kinases such as cyclinD/cdk4, cyclinE/cdk2, and cyclinA/cdk2 [39]. The overexpression of cyclins or the loss of expression of CDKIs is a significant factor in the high phosphorylation of the tumor suppressor Retinoblastoma (Rb) protein in cancer cells [41].

Although CDK-1 can substitute for CDK-2 in normal cells, CDK-2 becomes essential in malignant cells, facilitating cancer growth [36,42]. In the ongoing research, computational methodologies, including molecular docking, MD simulations, MM-GBSA, and ADME/T, have accelerated the discovery of CDK-2 inhibitors, providing a pathway to more selective and effective therapies [43]. Inhibitors such as sorafenib and aspirin have demonstrated potential in downregulating CDK-2 [44,45]; nevertheless, concerns regarding specificity and toxicity persist, requiring further research into more targeted therapies [46].

This study provides a thorough examination of CDK2 inhibitors, with a specific emphasis on the elucidation of their molecular mechanisms of action, pharmacological efficacy, and toxicity profiles. By leveraging cheminformatics approaches, such as molecular docking and MD simulations, this research identifies and characterizes promising lead compounds with high binding affinity and favorable pharmacokinetic properties. These findings contribute to the structure-based rational drug design pipeline, facilitating the development of selective and potent CDK2 inhibitors as potential therapeutic agents, particularly for the treatment of CDK2-driven malignancies such as colorectal cancer.

## 2. Methods and materials

**Fig 3:**
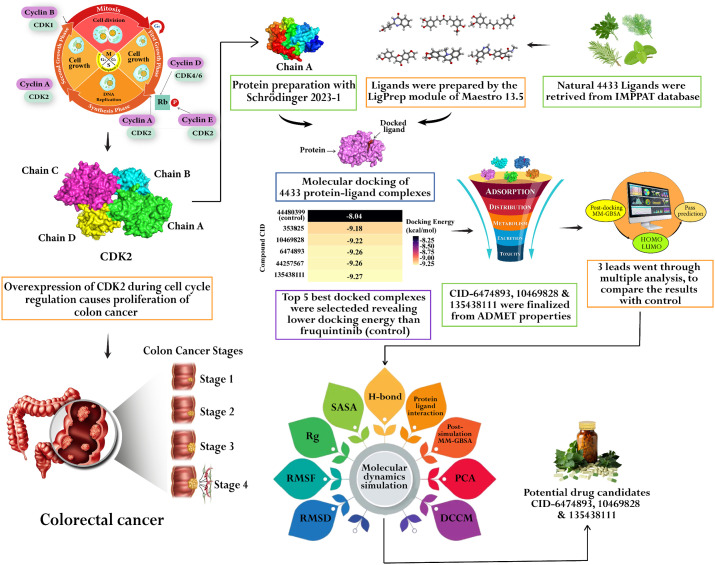
Workflow of the identification of phytochemicals that can effectively inhibit overexpressed CDK2 through the cheminformatics method.

The whole experimental framework is graphically presented in **Fig 3**.

### 2.1 Sourcing and pre-docking preparation of protein and ligand

**Fig 4:**
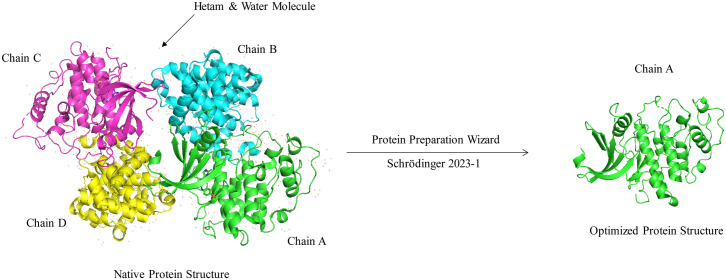
Chain A from the native CDK2 protein was isolated for molecular docking.

The crystal X-ray diffraction structure of CDK2 (PDB ID-6GUE), a protein that regulates the cell cycle in humans, was obtained from the RCSB Protein Data Bank (https://www.rcsb.org/) and shown in three dimensions. The X-ray diffraction method was employed to assess the structure of CDK2 with a resolution of 1.99 **Å** (generally referred to as < 2.5 **Å**). The R-value free was 0.234 [42]. The default Protein Preparation Wizard option of Schrödinger 2023–1 was used to remove chains (B, C, and D), ligands, hetero atoms, and water molecules as they often interrupt the docking process (**Fig 4**). The chain A-associated ligand was kept for the crystalline ligand-based grid box generation. The OPLS4 (Optimized Potentials for Liquid Simulations, version 4 extended) force field was utilized for prediction accuracy. The default parameter of the protein preparation wizard is used for assigning proper bond ordering, adding the H atom, and adding missing amino acid residues. Now, chain A is ready to be used effectively for screening and finding a lead compound for treating colorectal cancer.

The 3D conformation of the reference molecule, fruquintinib (CID-44480399), was obtained in SDF format from the PubChem database (https://pubchem.ncbi.nlm.nih.gov/). Fruquintinib is a therapeutic agent that inhibits VEGF for treating CRC [47]. A total of 4433 ligands were extracted from the IMPPAT database (https://cb.imsc.res.in/imppat/). Previous studies have shown that most plants have therapeutic action against human cervical, skin, colon, and lung cancer (**Supplementary** **S1**). Further utilization processes may include ligands prepared by the LigPrep module of Maestro 13.5, applying the OPLS4 force field, and decreasing ionization state by Epik at PH 7.

### 2.2 Molecular docking analysis

A computational approach to establish stable complexes between larger biomolecules (such as proteins, DNA, or RNA) and smaller molecules (such as ligands) is referred to as molecular docking [48]. The Schrödinger Protein Preparation Wizard was used to construct CDK2 (6GUE, chain A) to anticipate the best ligand-protein match via molecular docking studies. A grid box is created for the receptor, with the native ligand in the middle. The virtual screening was conducted by docking 4,433 compounds and controls with the target protein by using GLIDE available in Maestro v13.5 of Schrödinger, which led to the identification of the ligand exhibiting the highest docking score. The OPLS4 force field was employed in the docking process using standard precision mode. The Maestro viewer revealed the interacting chemical bonds that connect ligands and amino acid residues in the protein.

### 2.3 Pharmacokinetics and toxicity prediction

The interaction of pharmacokinetics, toxicity, and potency is essential for medication efficacy. The ADME/T profile of a compound ensures its safe and effective delivery to the target. Preliminary ADME/T assessment has diminished clinical trial failures due to inadequate pharmacological characteristics [49]. The control, along with the five top-scoring compounds from the docking analysis, was subjected to an in-depth evaluation of ADME properties and toxicity predictions to assess their pharmacokinetic and safety profiles.

To evaluate the ADME, physicochemical, drug-likeness, and pharmacokinetic properties of the chosen ligand compounds, the SwissADME online tool (http://www.swissadme.ch) was used [49]. Since small compounds have the potential to harm many human organs via cytotoxicity, carcinogenicity, hepatotoxicity, immunotoxicity, and mutagenicity, it is essential to assess their potential toxicity. Predicting toxicity is thus crucial to the medication development process. ProTox 3.0 (https://tox-new.charite.de/) was used in this investigation to estimate the toxicity profiles of the selected substances [50].

### 2.4 Post-docking MM-GBSA calculation

The MM-GBSA method was used to analyze the free binding energies of the protein and ligand complexes [51]. **Δ*G***_***bind***_** = *G***_***RL ***_**− *G***_***R***_** − *G***_***L***_ is the formula used to represent the binding energy of the ligand (L) to the receptor (R), which results in the complex (RL) [52]; a larger negative score denotes a bigger and longer binding energy [53]*.* The lead compounds with a lower docking score and those that successfully satisfied the ADME/T criteria were selected for further analysis using MM-GBSA computations. Maestro’s Prime MM-GBSA v3.00 software was used to calculate the MM-GBSA score. An indication for assessing the effectiveness of recently screened medications is the MM-GBSA score in conjunction with docking scores.

### 2.5 SAR analysis

The PASS online web tool (http://way2drug.com/PassOnline/predict.phpp) was used to evaluate the anticancer potential activity spectrum of the selected phytochemical substances and the control. The PASS program uses a database of more than 4,000 attributes to find bioactive chemicals with high prediction reliability, including drug-related and non-drug-related activities. The appropriate prediction findings are then expressed using both the probability of activity (**Pa**) and the probability of inactivity (**Pi**), which range from 0.00 to 1.00. A molecule must meet the requirement that **Pa > Pi**, which means that the total of **Pa** and **Pi** is equal to 1, to be deemed not completely inactive or active simultaneously [54]. The findings of the PASS prediction were used and understood in a flexible way, demonstrating that (i) when **Pa > 0.7**, there is a high probability of detecting the activity empirically, (ii) if **0.5 < Pa < 0.7**, there is a low probability of detecting the activity empirically, even though the chemical probably isn’t as comparable to existing pharmaceuticals, and (iii) if **Pa < 0.5**, there is a lower probability of detecting the activity empirically, but there is a probability of detecting a structurally similar pharmaceutical agent [55].

### 2.6 FMO analysis

The FMO analysis is regarded as a comprehensive method for examining the optoelectronic characteristics of a molecule [56]. The electronic properties of the selected compounds CIDs-6474893, 10469828, and 135438111 were examined by FMO analysis [57], focusing on the Highest Occupied Molecular Orbital (HOMO) and the Lowest Unoccupied Molecular Orbital (LUMO) [58]. The LUMO serves as an electron acceptor in molecular interactions, whereas the HOMO functions as an electron donor [59]. The Gaussian09 software [60] package was employed to conduct all calculations, and the output of these calculations was visualized by using the GaussView program [61]. The computations used the 6-31G basis set [62] and the Density Functional Theory (DFT) [63] approach with the restricted B3LYP (RB3LYP) [64] hybrid function. The HOMO–LUMO energy gap (**ΔE**), molecular hardness (**η**), and molecular softness (**S**) were determined to find out how reactive and stable the molecules are [65]. Parr and Pearson’s interpretation of DFT and Koopmans’ theorem was used to come up with these numbers, which link electron affinity (**A**) and ionization potential (**I**) to the energies of LUMO and HOMO orbitals [66,67]. The following equations were applied:

HOMO-LUMO energy gap, **ΔE = (E**_**LUMO**_ **– E**_**HOMO**_),

The energy of the HOMO is denoted as **E**_**HOMO**,_ and that of the LUMO as **E**_**LUMO**_.

Hardness, **η** **= (I-A)/2**

Softness, **S = 1/η**

A smaller **ΔE** value indicates greater reactivity and lower kinetic stability, while a larger **ΔE** value indicates greater stability and reduced reactivity [68,69]. Hardness (**η**) and softness (**S**) are global reactivity descriptors computed with the energies of the frontier molecular orbitals [65]. A more stable and less reactive molecule results from a larger Hardness (**η**) value [70]. Conversely, softness (**S**) indicates the capacity of the molecule to allow electrical alterations [71,72]. A reduced hardness (i.e., increased softness) indicates enhanced chemical reactivity and polarizability [73].

### 2.7 MD simulation

MD simulation is a highly effective approach for clarifying the interactions between potential pharmaceuticals and their biological targets [74]. Ligands and proteins may not regularly attain precise binding poses within the receptor pocket. Molecular Dynamics (MD) simulations can refine binding poses by employing force fields to assess dynamic interactions, facilitating modifications in ligand orientation to improve affinity and specificity. This procedure is essential for optimizing ligand design and enhancing therapeutic efficacy [75]. The Desmond module from Schrödinger was employed in a Linux environment to simulate molecular dynamics for 200 ns for this investigation [76]. The simple point-charge (SPC) water model was used to accurately characterize the membrane environment to guarantee appropriate electrical permittivity and water density. Na+ and Cl+ ions were used to neutralize the system and achieve a physiological salt concentration of 0.15 M. To keep the volume of a 10 × 10 × 10 **Å³** orthorhombic box constant, periodic boundary conditions were created. At 300 K and 1.01325 bar with 1.2 energy, molecular dynamics (MD) simulations were carried out in NPT ensemble conditions. Trajectories were recorded at 200 picosecond intervals to define interactions in the solvated protein-ligand system using the OPLS4 Force Field. By setting these settings, it was possible to accurately simulate the biological system and gain necessary knowledge about how proteins and ligands interact.

## 3. Results

### 3.1 Molecular docking analysis

**Fig 5:**
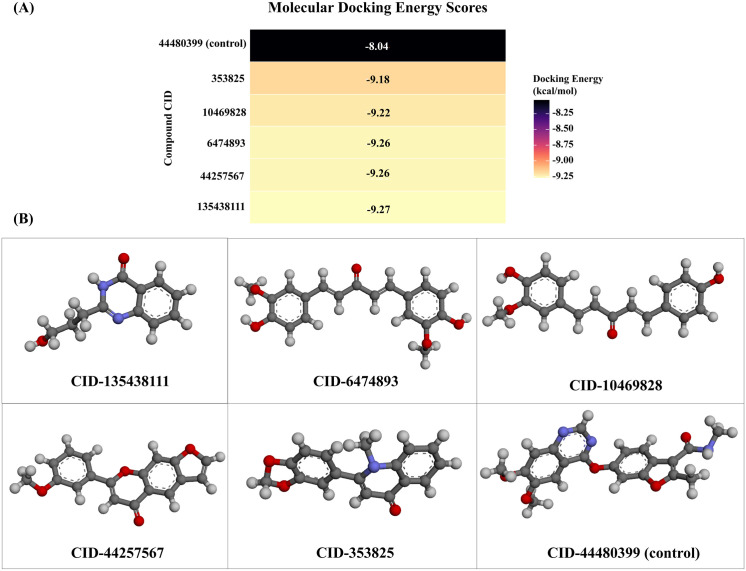
Molecular docking energy scores (A), 3D structures of the five best docked compounds (CID-135438111, 6474893, 10469828, 44257567, and 353825) and the control (CID-44480399) (B).

Molecular docking was used using 4433 drug-like compounds and the control drug fruquintinib (CID-44480399). Among the top five compounds with the highest docking energy, three had higher docking energy than fruquintinib (control). The top five compounds, which have a greater binding affinity towards the target, are CID-135438111 (IMPHY008184), CID-6474893 (IMPHY007097), CID-44257567 (IMPHY013402), CID-10469828 (IMPHY013402), and CID-353825 (IMPHY011367), compared to the control CID-44480399, as shown in **Fig 5**. Their docking scores are −8.037, −9.271, −9.255, −9.255, −9.218, and −9.176 kcal/mol, respectively (**Table 1**).

**Table 1:** Identification, docking energy score, source, and therapeutic applications of the top five chosen compounds and control (fruquintinib).

PubChem CID	Compound Name	Docking energy (kcal/mol)	Source (plant)	Therapeutic Use
135438111	Pegamine	−9.271	*Peganum harmala*	Anticancer Activities [77]
6474893	(1E,4E)-1,5-bis(4-hydroxy-3-methoxyphenyl) penta-1,4-dien-3-one	−9.255	*Curcuma longa*	Lung cancer [78,79]
44257567	Pongone	−9.255	*Pongamia pinnata*	MCF-7 breast cancer cells [80,81]
10469828	(1E,4E)-1-(4-Hydroxy-3-methoxyphenyl)-5-(4-hydroxyphenyl)-1,4-pentadien-3-one	−9.218	*Curcuma longa*	Lung cancer [78,79]
353825	Graveoline	−9.176	*Ruta graveolens*	Colon cancer [82,83]
44480399 (control)	Fruquintinib	−8.037	*N/A*	Colorectal cancer [84]

### 3.2 Pharmacokinetics and toxicity prediction

**Fig 6:**
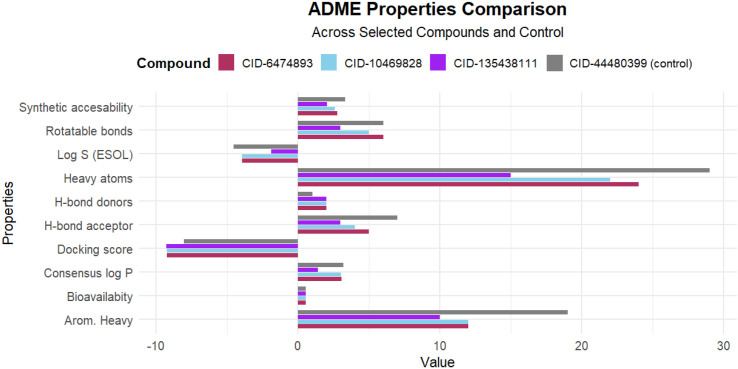
ADME properties of three lead compounds (CIDs-6474893, 10469828, and 135438111) and the control (CID-44480399).

**Fig 7:**
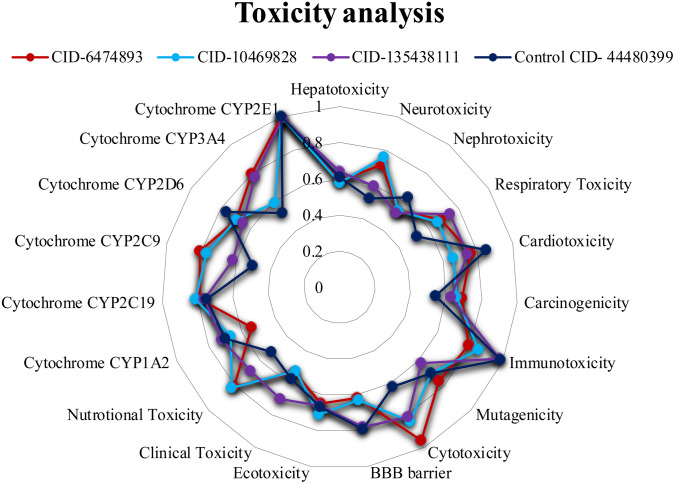
Toxicity properties of the three lead compounds (CIDs-6474893, 10469828, and 135438111) and the control (CID-44480399).

The drug’s pharmacokinetic profile can be improved by employing ADME properties, which are essential for comprehending the drug’s overall pharmacokinetics. Based on the docking scores of 4433 compounds, the top five phytochemical compounds (CID-135438111, 6474893, 44257567, 10469828, and 353825) and the control drug (CID-44480399) undergo ADME, pharmacokinetics, and toxicity properties analysis. From the inquiry, it was observed that three phytochemicals (CID-6474893, CID-10469828, and CID-135438111) showed druggable ADME, toxicity characteristics, and no Lipinski violations. Conversely, the control ligand (CID-44480399) exhibited active toxicity in various toxic properties, in comparison to the three lead compounds. Thus, the above three phytochemical compounds were selected for further analysis. The pharmacokinetics and toxicity properties of the three lead compounds (CIDs-6474893, 10469828, and 135438111) and the control (CID-44480399) are shown in Figs 6 and 7, respectively, and additional data are provided in **Supplementary** **S2**.

### 3.3 Protein-ligand interaction analysis

**Fig 8:**
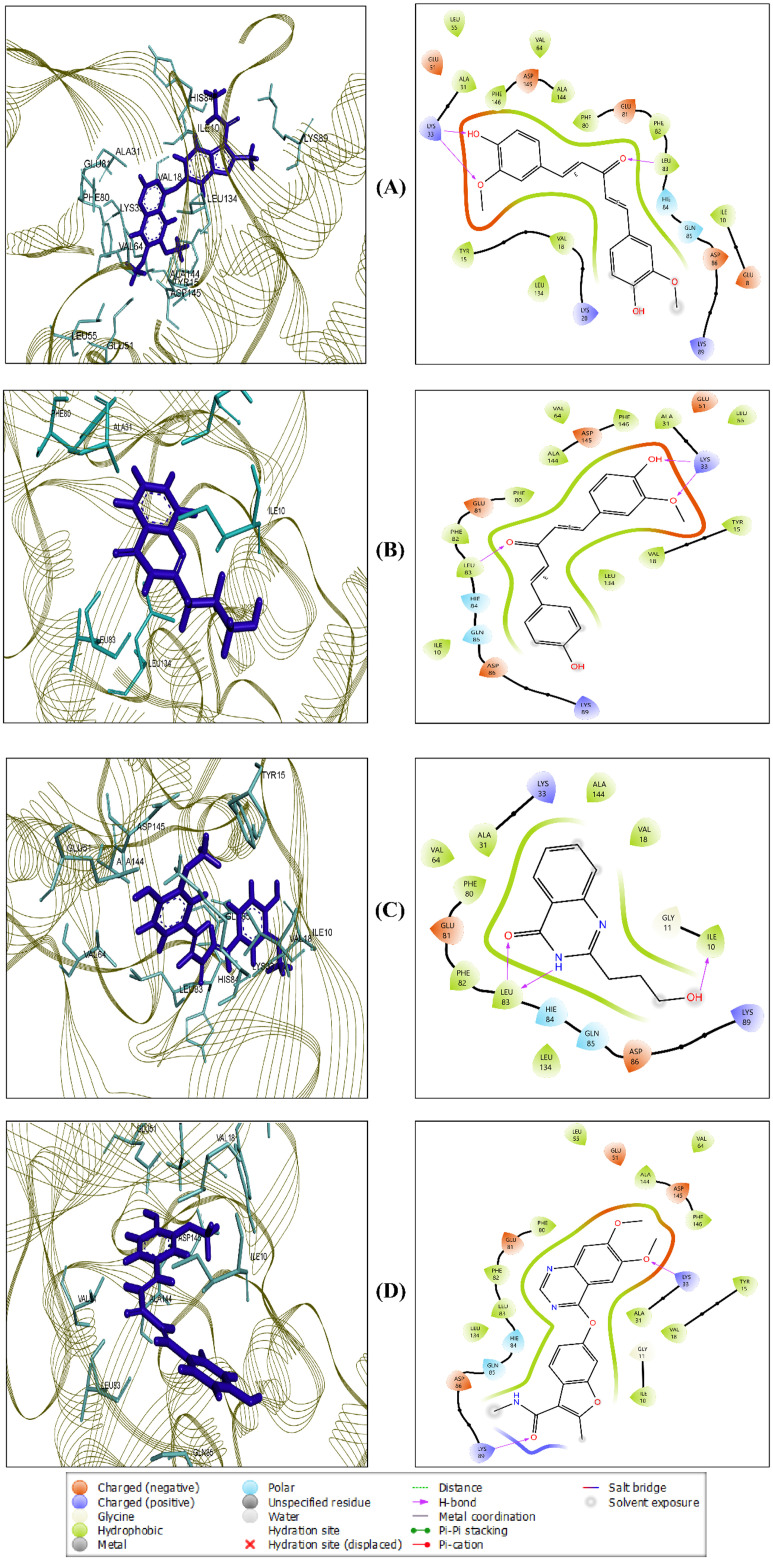
Protein-ligand interactions between the CDK2 protein and the three lead compounds, along with the control. CIDs-6474893 (A), 10469828 (B), 135438111 (C), and 44480399 (control) (D).

**Fig 9:**
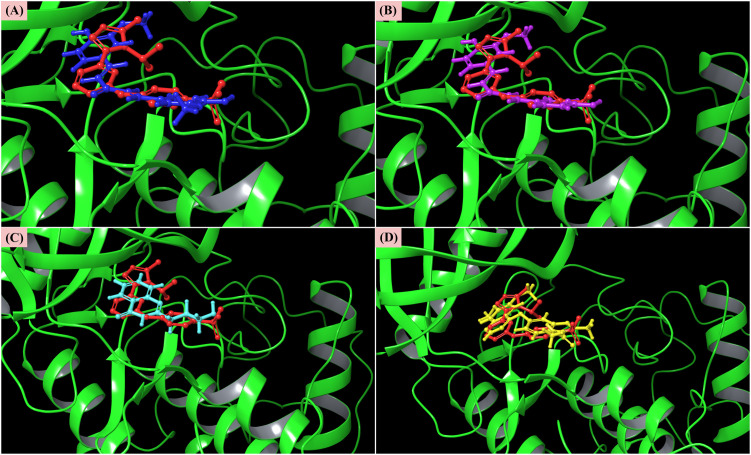
Validation of docking poses of selected ligands within the binding site of the target protein. The protein is represented in green cartoon format, with four docked ligands shown in different colors: CID-6474893 (blue, A), CID-10469828 (purple, B), CID-135438111 (cyan, C), and control CID-44480399 (yellow, D). All docked ligands are visualized in complex with the red-colored co-crystallized ligand to confirm accurate binding site alignment.

Based on binding affinity and ADME/T properties, three lead compounds, CIDs-6474893, 10469828, 135438111, and control CID-44480399, were chosen for further downstream analysis. Here, the maestro module of the Schrödinger suite was employed for visualizing molecular interactions. The interaction of amino acids with the three lead compounds and the control is shown in **Table 2** and depicted in **Fig 8**. Residues ILE_10, ALA_32, VAL_18, VAL_64, LEU_83, LEU_134, PHE_80, PHE_82, and ALA_144 were commonly involved in hydrophobic interactions. HIE_84 and GLN_85 frequently participated in polar interactions, while LYS_33 and LYS_89 were consistently involved in hydrogen bonding. These findings indicate that the three lead compounds and the control occupy the same binding pocket. The validation of docking poses of the three lead compounds, CIDs-6474893, 10469828, 135438111, and control CID-44480399 within the binding site of the target protein (CDK2) is displayed in **Fig 9**.

**Table 2:** Comprehensive analysis of intermolecular interactions and key amino acid residues involved in protein-ligand complex formation.

PubChem CID	Polar Bond	Hydrophobic Bond	Hydrogen Bond
6474893	HIE_84, GLN_85	ALA_31, ILE_10, TYR_15, VAL_18, LEU_55, VAL_64, PHE_80, PHE_82, PHE_146, LEU_83, LEU_134, ALA_144	LYS_20, LYS_33, LYS_89
10469828	HIE_84, GLN_85	VAL_18, VAL_64, ILE_10, TYR_15, ALA_31, LEU_55, PHE_82, LEU_83, LEU_134, ALA_144, PHE_146, PHE_80	LYS_33, LYS_89
135438111	HIE_84, GLN_85,	ALA_144, VAL_18, VAL_64, PHE_80, LEU_134, LEU_83, ILE_10, ALA_31, PHE_82	LYS_33, LYS_89
44480399 (control)	HIE_84, GLN_85	TYR_15, LEU_55, PHE_80, VAL_18, LEU_83, ALA_31, LEU_134, VAL_64, ALA_144, PHE_82, PHE_146, ILE_10	LYS_33, LYS_89

### 3.4 Post-docking MM-GBSA calculation

**Fig 10:**
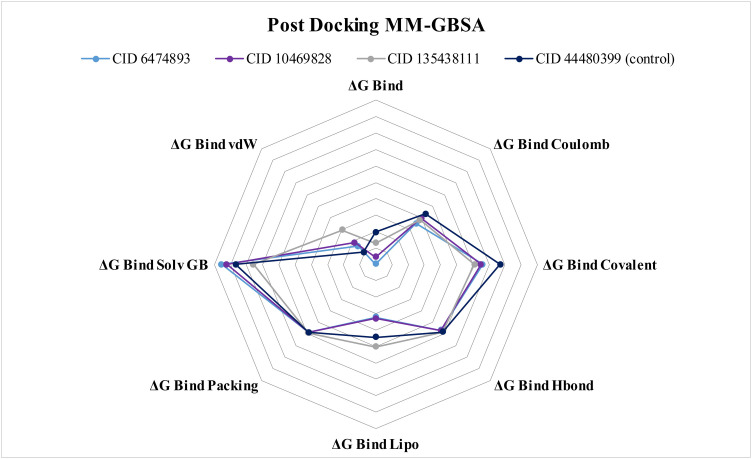
The radar chart shows the post-docking MM-GBSA scores of the three lead compounds (CIDs-6474893, 10469828, and 135438111) and the control (CID-44480399).

The MM-GBSA process was used for calculating the binding free energy between the protein and ligand. The target protein CDK-2 interacted with the control medication Fruquintinib and the three lead compounds (CIDs-6474893, 10469828, and 135438111) with higher docking scores. The examination of the MM-GBSA result revealed that they had an increased net negative pattern and higher binding free energy scores. The MM-GBSA results signify that the leads CIDs-6474893, 10469828, 135438111, and the control drug CID-44480399 showed negative binding free energies of −59.79, −55.64, −46.99, and −40.39 kcal/mol, respectively. The lead compounds demonstrated a more stable association with the target protein than the control medication, with CID-6474893 exhibiting the most significant binding energy release (−59.79 kcal/mol), as illustrated in **Fig 10**. Further study of these three compounds revealed significant activity in ΔG Bind Covalent, ΔG Bind Coulomb, ΔG Bind Lipo, ΔG Bind Hbond, ΔG Bind Packing, ΔG Bind Solv GB, and ΔG Bind vdW (**Fig 10** and Supplementary S3).

### 3.5 SAR analysis

The chemistry and physical structure of a phytoconstituent significantly influence its biological functions. The physiological activities of medicinal plants typically support their use, which is crucial for evaluating their potential as therapeutic candidates. A SAR analysis was performed utilizing the web-based PASS tool to assess the anticancer effectiveness of the chosen compounds in conjunction with the control fruquintinib, before the MD simulation. The findings of the SAR models are illustrated in Table 3 and Supplementary S4. The three lead compounds possess a biological function associated with anti-cancer activity according to this analysis. The initial lead compound (CID-6474893) exhibited antimutagenic, antineoplastic, and apoptosis agonist properties. The second lead (CID-10469828) exhibited properties such as antineoplastic, antimutagenic, TP53 expression, apoptosis agonist, enhancement, and TNF expression inhibition. Lastly, the third lead compound (CID-135438111) demonstrated TP53 expression enhancement, antineoplastic properties, chemosensitization, radiosensitization, and immunomodulatory activity for the treatment of cancer-associated disorders. These numerous prior biological activities can guarantee that the selected three compounds will undergo a more thorough evaluation. On the other hand, the control ligand (CID-44480399) revealed many properties as well, such as an angiogenesis inhibitor, antineoplastic, vascular endothelial growth factor antagonist, proto-oncogene tyrosine-protein kinase Fgr inhibitor, platelet-derived growth factor receptor kinase inhibitor, and so on.

**Table 3:** Predicted biological activities of selected compounds and the control ligand with corresponding Pa and Pi values.

Compound CID	SMILES	Pa	Pi	Activity
6474893	COC1 = C(C = CC(=C1)/C = C/C(=O)/C = C/C2 = CC(=C(C = C2)O)OC)O	0.926	0.004	HIF1A expression inhibitor
		0.916	0.001	Preneoplastic conditions treatment
		0.906	0.010	Membrane integrity agonist
		0.889	0.007	Mucositis treatment
		0.877	0.005	Apoptosis agonist
		0.841	0.008	Antineoplastic
		0.838	0.011	Mucomembranous protector
		0.823	0.003	Antimutagenic
		0.816	0.004	Prostate cancer treatment
		0.752	0.020	Membrane permeability inhibitor
		0.717	0.023	TP53 expression enhancer
10469828	COC1 = C(C = CC(=C1)/C = C/C(=O)/C = C/C2 = CC = C(C = C2)O)O	0.930	0.004	HIF1A expression inhibitor
		0.922	0.001	Preneoplastic conditions treatment
		0.915	0.008	Membrane integrity agonist
		0.892	0.007	Mucositis treatment
		0.877	0.003	TNF expression inhibitor
		0.874	0.005	Apoptosis agonist
		0.842	0.003	Antimutagenic
		0.840	0.008	Antineoplastic
		0.837	0.011	Mucomembranous protector
		0.813	0.004	Prostate cancer treatment
		0.806	0.003	MAP kinase stimulant
		0.764	0.017	Membrane permeability inhibitor
		0.735	0.019	TP53 expression enhancer
		0.711	0.004	Free radical scavenger
135438111	C1 = CC = C2C(=C1)C(=O)NC(=N2)CCCO	0.307	0.084	Antineoplastic (solid tumors)
		0.392	0.009	Antineoplastic enhancer
		0.315	0.136	Chemosensitizer
		0.302	0.136	Radiosensitizer
		0.398	0.142	TP53 expression enhancer
		0.418	0.088	Preneoplastic conditions treatment
		0.318	0.073	Cancer-associated disorders treatment
44480399 (control)	CC1 = C(C2 = C(O1)C = C(C = C2)OC3 = NC = NC4 = CC(=C(C = C43)OC)OC)C(=O)NC	0.818	0.005	Angiogenesis inhibitor
		0.654	0.034	Antineoplastic
		0.623	0.005	Vascular endothelial growth factor antagonist
		0.576	0.008	Antineoplastic (solid tumors)
		0.538	0.005	Vascular endothelial growth factor 2 antagonist
		0.437	0.053	Platelet-derived growth factor receptor kinase inhibitor
		0.358	0.025	Proto-oncogene tyrosine-protein kinase Fgr inhibitor
		0.367	0.085	Nucleotide metabolism regulator
		0.332	0.134	Antiinflammatory

### 3.6 FMO analysis

**Fig 11:**
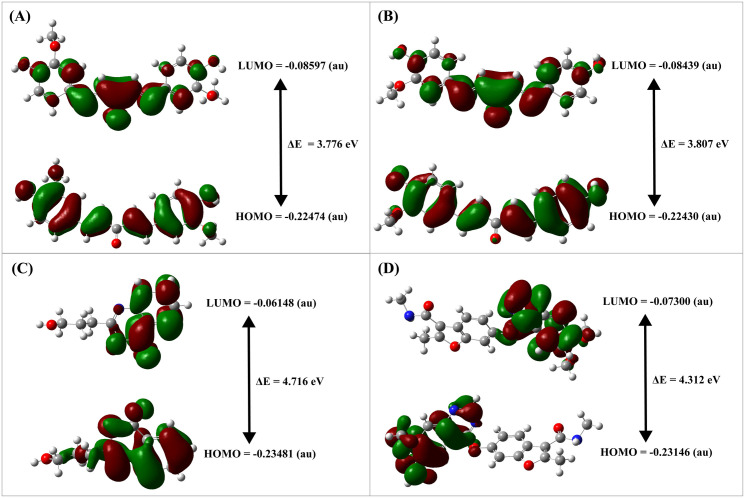
HOMO-LUMO Gap (ΔE), HOMO, and LUMO of the three lead compounds CIDs-6474893. (A), 10469828 (B), 135438111 (C), and control 44480399 (D).

The three lead compounds of CIDs-6474893, 10469828 and 135438111, and the control Fruquintinib CID-44480399 exhibited a HOMO and LUMO energy score of (−0.22474; −0.08597), (−0.22430; −0.08439), (−0.23481; −0.06148), and (−0.23146; −0.07300), respectively in atomic mass units (au) that are showed at Fig 11 and Table 4. The calculated HOMO-LUMO energy gap (**ΔE**), molecular hardness (**η**), and molecular softness (**S**) energy scores of the three leads and control were (3.776, 3.807, 4.716, and 4.312) eV, (1.888, 1.9035, 2.358, and 2.156) eV, and (0.530, 0.525, 0.424, and 0.464) eV^-1^, respectively. From the analysis of this investigation, it can be stated that CID-6474893 had the highest chemical reactivity, most responsive to electron transfer, and least resistant to electron density change, as evidenced by its lowest HOMO-LUMO energy gap (**ΔE**), highest softness (**S**), and lowest hardness (**η**). On the other hand, CID-135438111 had the largest **ΔE**, highest **η**, and lowest **S**, indicating low chemical reactivity, resistance to change in electron density, and poor responsiveness to interactions. Conversely, CID-10469828 revealed moderate energy scores in **ΔE**, **η**, and **S**, explaining its balanced interaction and chemical reactivity. Comparing the three leads with the control, CID-6474893 and 10469828 are more likely to engage in effective electronic interactions.

**Table 4:** HOMO-LUMO energy gap (ΔE), molecular hardness(η), and molecular softness (S) energy score of the three lead compounds CID-6474893, 10469828, 135438111, and the control 44480399.

Compounds CID	HOMO (au)	LUMO (au)	HLG (au)	HLG (eV)	I (-E_HOMO_) (au)	I (eV)	A (-E_LUMO_) (au)	A (eV)	η (eV)	S (eV^-1^)
6474893	−0.22474	−0.08597	0.13877	3.776	0.22474	6.115	0.08597	2.339	1.888	0.530
10469828	−0.22430	−0.08439	0.13991	3.807	0.22430	6.103	0.08439	2.296	1.9035	0.525
135438111	−0.23481	−0.06148	0.17333	4.716	0.23481	6.389	0.06148	1.673	2.358	0.424
44480399 (control)	−0.23146	−0.07300	0.15846	4.312	0.23146	6.298	0.07300	1.986	2.156	0.464

### 3.7 MD simulation analysis

The MD simulation was run for 200 ns, utilizing the Desmond package from Schrödinger. The Simulation Interaction Diagram (SID) was then used to examine the RMSD, RMSF, Rg, SASA, protein-ligand interactions, and ligand-protein interactions. Maestro’s simulation event analysis option was utilized to analyze H-bonds. Using the R programming (Bio3D package), PCA and DCCM were determined from the MD trajectories. Besides that, post-simulation MM-GBSA were also performed on the MD trajectories in the initial and terminating stages (0 ns and 200 ns) using the Prime MM-GBSA module of Desmond.

#### 3.7.1 Protein RMSD analysis.

**Fig 12:**
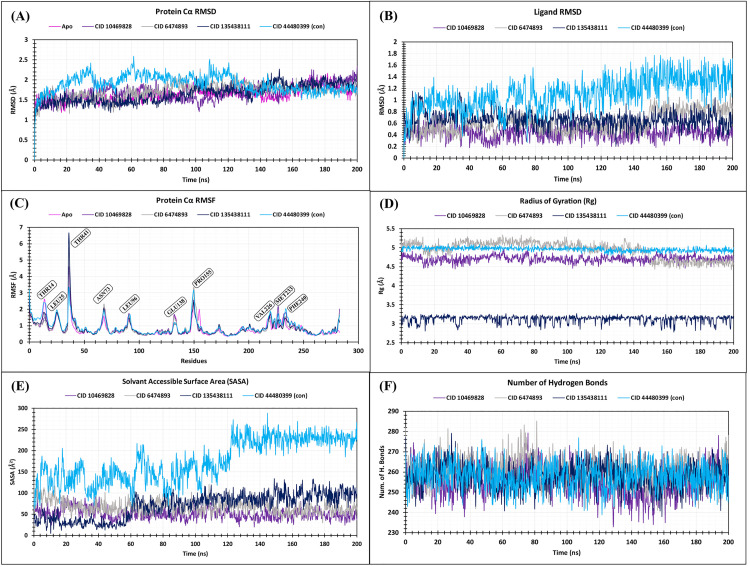
Protein and ligand RMSD, RMSF, Rg, SASA, and H-bonds of the ligand-protein complex of CIDs-6474893, 10469828, 135438111, and 44480399 (control), respectively (A, B, C, D, E, F).

RMSD is frequently applied to establish the equilibration period, quality of biomolecular simulations, and to group conformations that are comparable in MD trajectory analysis [85]. The acceptable range of protein-ligand complex’s average RMSD change is between 1 and 3 **Å**. If the RMSD value is more than 1–3 **Å**, there has been a significant conformational change in the protein structure [86]. The RMSD of the three lead compounds and the control has been assessed to identify alterations in protein structure from the initial state. A 200 ns MD simulation was performed to investigate the conformational change of the target macromolecule within the complex of the specified chemicals, including CIDs-6474893, 10469828, 135438111, and 44480399 (control), shown in Table 5, Fig 12A, and Supplementary S5. The highest RMSD values of the apoprotein, selected three lead compounds (CIDs-6474893, 10469828, and 135438111) and control (CID-44480399), were 2.16, 2.27, 2.348, 2.264, and 2.587 **Å**, respectively, and the lowest scores were 1.187, 0.993, 1.003, 1.095, and 0.934 **Å**, respectively. The average RMSD scores for apoprotein, three lead compounds (CIDs-6474893, 10469828, and 135438111), and control (CID-44480399) were 1.628, 1.715, 1.683, 1.653, and 1.9 **Å**, respectively. The average RMSD values of all substances remained within the acceptable range of 1–3 **Å**. The control (CID-44480399) exhibited excessive fluctuation during the 20–40, 40–80, and 100–120 ns simulation time ranges and failed to maintain a constant fluctuation with the apoprotein throughout the 20–120 ns simulation time range. Compared to the control (CID-44480399), all three lead compounds (CIDs-6474893, 10469828, and 135438111) worked very well when mixed with apoprotein till the conclusion of the simulation session and kept a steady level of fluctuation, as their average RMSD values were less than that of the control.

**Table 5:** Three lead compounds, together with the control, produced distinct parameters, such as the greatest, smallest, and average values from the 200 ns MD simulation.

Parameter	Value	Apo	CID 6474893	CID 10469828	CID 135438111	CID 44480399 (Control)
Protein Cα RMSD	H. RMSD (Å)	2.16	2.28	2.35	2.26	2.59
	L. RMSD (Å)	1.19	0.99	1.00	1.09	0.93
	A. RMSD (Å)	1.63	1.72	1.68	1.65	1.9
Ligand RMSD	H. RMSD (Å)	N/A	1.12	0.78	1.16	1.76
	L. RMSD (Å)	N/A	0.25	0.17	0.20	0.27
	A. RMSD (Å)	N/A	0.61	0.43	0.66	1.10
Protein Cα RMSF	H. RMSF (Å)	4.51	5.67	4.52	6.57	3.32
	L. RMSF (Å)	0.34	0.36	0.36	0.36	0.36
	A. RMSF (Å)	0.84	0.88	0.90	0.90	0.95
Radius of gyration	H. Rg(Å)	N/A	5.33	4.98	3.27	5.09
	L. Rg (Å)	N/A	4.42	4.43	2.79	4.80
	A. Rg (Å)	N/A	4.94	4.70	3.12	4.96
Solvent-accessible surface area	H. SASA (Å^2^)	N/A	126.49	93.98	134.05	288.34
	L. SASA (Å^2^)	N/A	30.12	19.97	5.21	62.86
	A. SASA (Å^2^)	N/A	67.67	51.26	69.88	175.58
Hydrogen bond	H. H-bond	N/A	285	279	279	277
	L. H-bond	N/A	241	233	238	239

#### 3.7.2 Ligand RMSD analysis.

Ligand RMSD was evaluated to demonstrate the stability of the ligand while associated with the protein binding site. Lower ligand RMSD indicates better stability of a molecule [87]. The ligand RMSD analysis of the three lead compounds (CIDs-6474893, 10469828, and 135438111) and the control (CID-44480399) were shown in Table 5, Fig 12B, and Supplementary S6. According to the results, three lead compounds (CIDs-6474893, 10469828, and 135438111) showed a steadier range of variations, compared to the control, with the lowest and highest values of 0.25 **Å** in the 42 no. frame to 1.117 **Å** in the 993 no. frame (the range is 0.867 **Å**), 0.167 **Å** in the 260 no. frame to 0.777**Å** in the 854 no. frame (the range is 0.61 **Å**), 0.201 **Å** in the 645 no. frame to 1.156 **Å** in the 26 no. frame (the range is 0.955 **Å**), respectively. Consequently, the control displayed a higher fluctuation with a lowest and highest value of 0.269 **Å** in the 375 no. frame to 1.764 **Å** in the 781 no. frame (the range is 1.495 **Å**). The average ligand RMSD scores for three lead compounds (CIDs-6474893, 10469828, and 135438111), along with the control (CID-44480399), were 0.613 **Å**, 0.432 **Å**, 0.664 **Å**, and 1.102 **Å**, accordingly. All three lead compounds (CIDs-6474893, 10469828, and 135438111) showed lower average ligand RMSD compared to the control (CID-44480399), and among the three lead compounds, CID-10469828 showed the lowest average ligand RMSD, suggesting that CID-10469828 has greater stability than the other compounds (CIDs-6474893 and 135438111). Besides this fact, the difference among the average RMSD values of the three lead compounds was not much, and thus all three of them can be declared as stable compounds compared to the control (CID-44480399).

#### 3.7.3 RMSF analysis.

Utilizing the RMSF approach, the change in protein chain residues and the positioning of ligand atoms can be estimated. The RMSF value of the AA (amino acid) atoms reveals how stable and how much they change in a complicated system. If a compound’s AA residues have low RMSF values, it may be more stable. If a complex’s AA residues have high RMSF values, it may be less stable [86]. The RMSF values of the three lead compounds (CIDs-6474893, 10469828, 135438111) and control (CID-44480399), in complex with the apoprotein, are shown in Table 5, Fig 12C, and Supplementary S7. The greatest RMSF score of apoprotein, three lead compounds (CIDs-6474893, 10469828, and 135438111), and control (CID-44480399) were 4.511, 5.673, 4.516, 6.569, and 3.321 **Å**, respectively, and the smallest values were 0.337, 0.355, 0.358, 0.364, and 0.362 **Å**, respectively. The average RMSF values for apoprotein, three lead compounds (CIDs-6474893, 10469828, and 135438111), and control (CID-44480399) were 0.836, 0.881, 0.897, 0.897, and 0.951 **Å**, respectively. All three lead compounds (CIDs-6474893, 10469828, and 135438111) showed smaller average RMSF compared to the control (CID-44480399). The chosen compounds exhibited a peak area of the protein at THR_14, LEU_25, THR_41, ASH_73, LEU_96, GLU_138, PRO_155, VAL_226, MET_233, and PHE_240 residual sites that showed the greatest fluctuation over the simulation period, as shown in **Fig 12C**.

#### 3.7.4 Rg analysis.

The Rg may be thought of as the arrangement of atoms along the protein-ligand complex axis. To determine the protein’s stiffness and mobility, the protein-ligand complexes’ Rg were analyzed [86]. A protein molecule with a lower Rg value is said to be closely packed, while one with a larger Rg value is said to be loosely packed [88]. The three lead compounds (CIDs-6474893, 10469828, and 135438111) and the control (CID-44480399) linked to the target protein were screened for stability in terms of Rg throughout the 200 ns simulation period, shown in Table 5, Fig 12D, and **Supplementary** **S6**. According to the results, three lead compounds (CIDs-6474893, 10469828, and 135438111) and the control (CID-44480399) showed a range of variations, with the lowest and highest values of 4.422 **Å** in the 993 no. frame to 5.327 **Å** in the 305 no. frame (the range is 0.905 **Å**), 4.434 **Å** in the 162 no. frame to 4.984 **Å** in the 309 number frame (the range is 0.55 **Å**), 2.786 **Å** in the 820 no. frame to 3.267 **Å** in the 528 no. frame (the range is 0.481 **Å**), and 4.803 **Å** in the 631 no. frame to 5.085 **Å** in the 141 no. frame (the range is 0.282 **Å**). The average Rg score for three lead compounds (CIDs-6474893, 10469828, and 135438111), along with the control (CID-44480399), were 4.939, 4.704, 3.123, and 4.963 **Å**, respectively. According to the result of this investigation, all three lead compounds (CIDs-6474893, 10469828, and 135438111) had lower Rg values compared to the control, but CID**-**135438111 had the lowest Rg value, indicating that CID-135438111 had the highest stability during the simulation period. These outcomes determine that the protein’s active site fails to undergo significant structural modification when binding to the lead compounds. Meanwhile, the control (CID-44480399) exhibited conformational changes in the protein’s active site. As the lead compounds (CIDs-6474893, 10469828, and 135438111) had a lower Rg value, suggesting that they were tightly packed with the protein, other than the control (CID-44480399). Additionally, CID-135438111, displaying the lowest Rg value, did not fluctuate at all during the simulation time, while the control (CID-44480399) revealed the most notable fluctuation throughout the simulation.

#### 3.7.5 SASA analysis.

The SASA has developed to comprehend the architecture and role of biological macromolecules. On a protein’s surface, the amino acid residues generally serve as active sites and/or interact with other molecules. Analysis of the SASA assists in identifying the solvent-like characteristics (hydrophobic or hydrophilic) of a protein molecule and protein-ligand complexes [86]. A molecule exhibiting a reduced solvent-accessible surface area (SASA) value is tightly compacted with few exposed surfaces, while an elongated or stretched molecule has a greater SASA value [89]. The corresponding SASA values of CIDs-6474893, 10469828, 135438111, and 44480399 (control) were calculated and displayed in Table 5, Fig 12E, and Supplementary S6. According to Fig 12E, three lead compounds (CIDs-6474893, 10469828, and 135438111) showed a steadier range of variations, compared to the control (CID-44480399), with the lowest and highest values of 30.121 **Å**^**2**^ in the 894 no. frame to 126.486 **Å**^**2**^ in the 1 no. frame (the range is 96.365 **Å**^**2**^), 19.969 **Å**^**2**^ in the 682 no. frame to 93.982 **Å**^**2**^ in the 104 no. frame (the range is 74.013 **Å**^**2**^), 5.213 **Å**^**2**^ in the 52 no. frame to 134.051 **Å**^**2**^ in the 744 no. frame (the range is 128.838 **Å**^**2**^), respectively. Consequently, the control displayed a higher fluctuation with the lowest and highest value of 62.859 **Å**^**2**^ in the 1 no. frame to 288.345 **Å**^**2**^ in the 723 no. frame (the range is 225.486 **Å**^**2**^). The three lead compounds (CIDs-6474893, 10469828, and 135438111), along with the control (CID-44480399), had respective average SASA values of 67.673, 51.263, 69.879, and 175.577 **Å**^**2**^. According to this investigation, the reduced average SASA score of the three lead compounds (CIDs-6474893, 10469828, and 135438111) indicates that they are binding in the backbone of the protein, occupying less surface area and more compact binding than the control (CID-44480399). Among the three leads, the CID-10469828 is slightly better, as evidenced by the lowest SASA value, indicating it is more profoundly entrenched inside the protein’s binding pocket in the complex system, hence augmenting its efficacy in regulating the biological activity of the target.

#### 3.7.6 Hydrogen bond analysis.

A drug-binding site that is actively involved in the interaction between the drug and the desired protein and that influences drug adsorption, specificity, and metabolism can be characterized by hydrogen bonds [90]. Therefore, the number of hydrogen bonds of the selected three lead compounds (CIDs-6474893, 10469828, and 135438111), compared with the control (CID-44480399), has been calculated for systems by looking at conformations every 200 ps, as displayed in Table 5, Fig 12F, and Supplementary S6. During the 200 ns simulation period, all compounds concurrently formed numerous hydrogen bonds, with distances varying from 233 to 285. The lowest and highest number of H-bonds of the three lead compounds (CIDs-6474893, 10469828, and 135438111) and the control (CID-44480399) were 241 to 285, 233 to 279, 238 to 279, and 239 to 277, respectively. The average number of H-bonds for the three lead compounds (CIDs-6474893, 10469828, and 135438111) and control (CID-44480399) were 260.676, 255.337, 258.011, and 257.905, respectively. According to the outcome, CID-6474893 and 135438111 had a larger average number of H-bonds than the control (CID-44480399), suggesting that they are more likely to establish hydrogen bonds more forcefully when they bind to the target protein. This could potentially improve drug selectivity.

#### 3.7.7 Protein-ligand interaction analysis.

**Fig 13:**
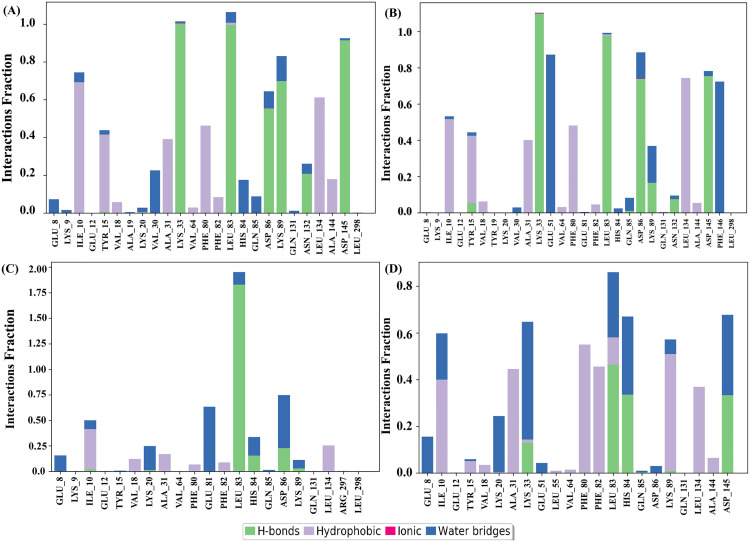
The protein–ligand interface’s diverse bonding patterns are demonstrated during the 200 ns MD simulation. The three lead compounds, CIDs-6474893 (A), 10469828 (B), and 135438111 (C), as well as the control, CID-44480399 (D), are displayed.

**Fig 14:**
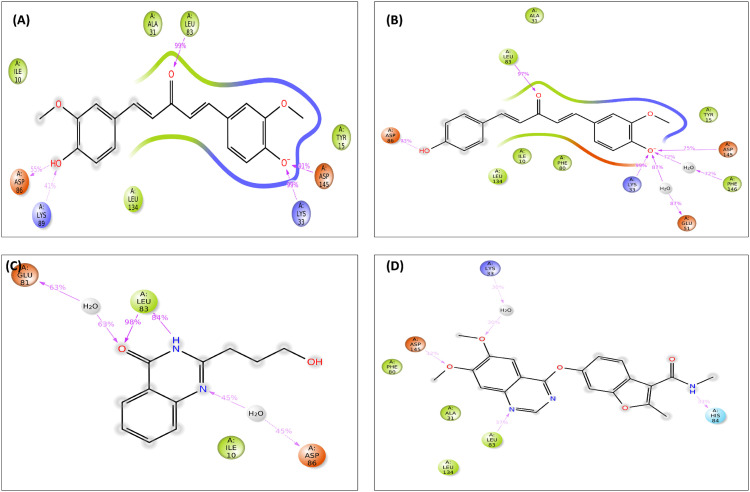
2D interactions of the ligand–protein complex after a 200 ns simulation. The three lead compounds and control: CIDs-6474893 (A), 10469828 (B), 135438111 (C), and 44480399 (D) are presented here.

The SID was used to investigate a protein’s complex structure, assign ligands, and examine interactions between molecules over the 200 ns simulation time. Non-covalent (hydrophobic bonds), ionic, hydrogen bonds, and water bridges were utilized to study and show the protein-ligand complexes. The 200 ns simulation ended with a stable binding with the target protein, as each molecule interacted with it in a different way. CID-6474893 produced more than two contacts at the residues TYR_15, LYS_20, LYS_33, LEU_83, ASP_86, and LYS_89, with interaction fractions (IF) of 0.44, 0.03, 1.02, 1.1, 0.65, and 0.85, respectively (Fig 13A). In Figs 13 and 14, the simulation yields a robust specific interaction as the identical subtype engages with the ligand repeatedly. The residues TYR_15 (0.47), LYS_33 (1.1), LEU_83 (1.0), and ASP_86 (0.9) produced multiple contacts with CID-10469828 (**Fig 13B**). CID-135438111 also made multiple interactions at residues ILE_10 (0.5) and ASP_86 (0.75) (**Fig 13C**). The control (CID-44480399) produced various contacts at the residues, LYS_20 (0.25), LYS_33 (0.65), LEU_83 (0.85), LYS_89 (0.53) (**Fig 13D**). Among the selected compounds (CIDs-6474893, 10469828, and 135438111) and the control (CID-44480399), the desired lead compound (CID-6474893) produced multiple contacts at the residues with a higher interaction fraction, suggesting that it is showing promising interaction with the target protein.

#### 3.7.8 Ligand-protein interaction analysis.

All three lead compounds (CIDs-6474893, 10469828, and 135438111) and the control (CID-44480399) have been shown to interact with one another in the protein space of the SID. When compared to alternative lead compounds (CIDs-10469828 and 135438111), the desired compound (CID-6474893) exhibited the highest stability due to its strong hydrogen bonding network, hydrophobic interactions, and engagement with key amino acid residues during the simulation. Accordingly, repeated interactions with the same kind of ligand maintain the distinct interaction (**Fig 14**). In comparison to the control (CID-44480399), compound CID-6474893 showed improved stability in the ligand-protein interaction investigation.

#### 3.7.9 Post-simulation MM-GBSA Analysis.

**Fig 15:**
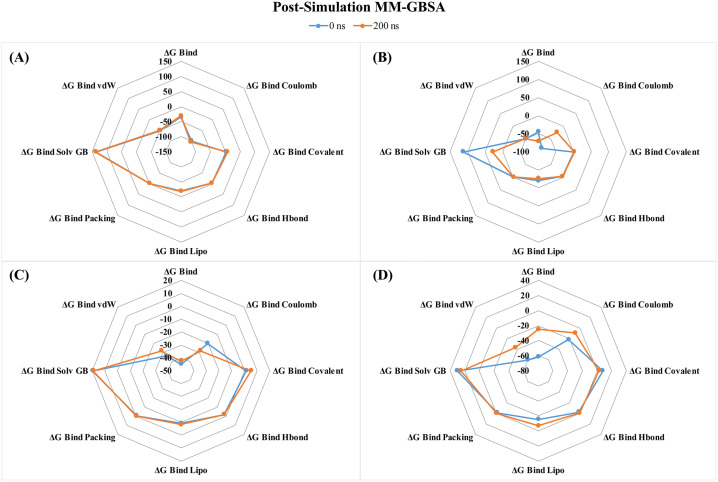
Post-simulation MM-GBSA analysis of the three lead compounds and the control: CIDs-6474893 (A), 10469828 (B), 135438111 (C), and 44480399 (D).

In this investigation, the MM-GBSA approach was used to determine the target protein ligand-binding free energy, as shown in **Fig 15**. In order to determine the MM-GBSA of the protein-ligand complex structure, the 200-ns dynamic simulation trajectory was used. The negative binding free energy (ΔG Bind) values of the three lead compounds (CIDs-6474893, 10469828, and 135438111) and the control (CID-44480399) in complex with the targeted protein were (−34.43 and −30.66), (−45.16 and −71.11), (−44.91 and −42.70), and (−61.79 and −24.94) kcal/mol, respectively, at the beginning and end of the experiment (200 ns) (Fig 15 and Supplementary S8). These ΔG Bind values represent the evolution of their stability during molecular dynamics (MD) simulations. The control exhibited greater stability at the outset, but it experienced a substantial decline in stability over time, indicating a weakened binding in the long term. As a result, CID-6474893 maintained a relatively stable state, with only a minor increase in free energy, but a lower stability reduction than the control. Conversely, CID-135438111 maintained a virtually identical level of stability, with only a minor increase in free energy. However, CID-10469828 demonstrated a substantial increase in stability, with a significant decrease in free energy during the simulation, suggesting that it has a superior binding affinity compared to the control and other lead compounds.

#### 3.7.10 PCA.

**Fig 16:**
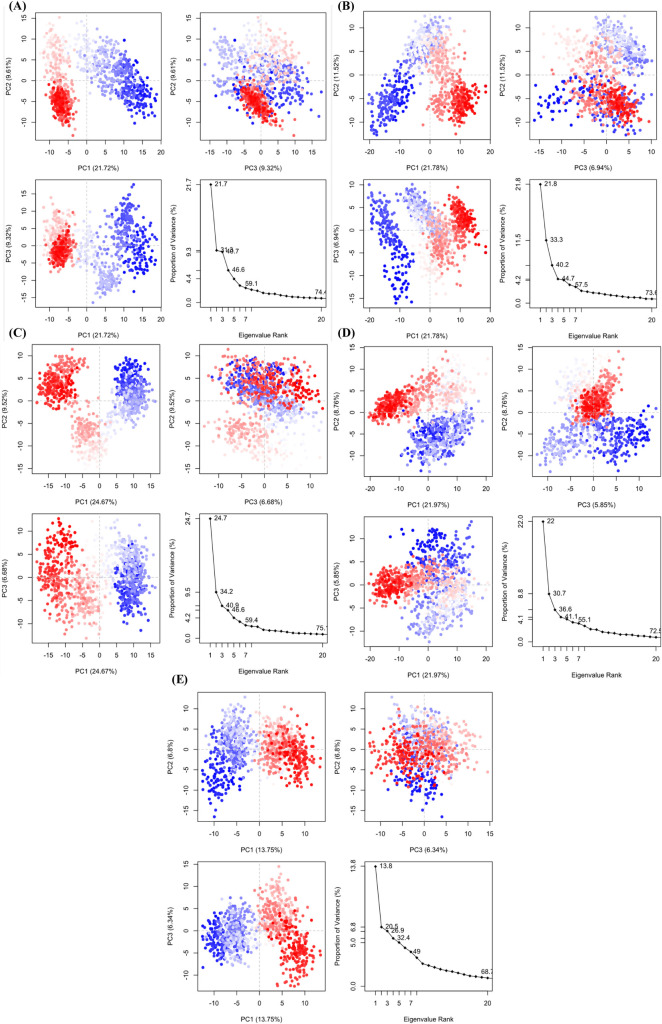
Comparing the eigenvalue using PCA and the variance percentage. Each area is shown on one of three panels. PC1, PC2, and PC3 are three variants. At this location, CIDs-6474893 (A), 10469828 (B), 135438111 (C), control 44480399 (D), and apoprotein (PDB ID-6GUE) (E).

PCA was conducted on the trajectory data of the three lead compounds CIDs-6474893, 10469828, 135438111, the control 44480399, and the apoprotein (PDB ID-6GUE) to examine the protein-ligand complexes’ conformational dynamics during MD simulations. The dynamics and structures of the complexes were identified by analysing the first three principal components (PCs) [88]. CID-6474893 (**Fig 16A**) had 21.72% variance from PC1, 9.61% from PC2, and 9.32% from PC3, totaling 40.65%. A significant categorization of conformations along these PCs shows considerable conformational differences across states. The eigenvalue scree plot shows a progressive decrease in variance contribution, implying a dispersed component motion contribution. In comparison, for CID-10469828 (**Fig 16B**), PC1 contributed 21.78% of the variance, followed by PC2 (11.52%) and PC3 (6.94%), accounting for a total variance of 40.24%. Conversely, CID-135438111 (**Fig 16C**) displayed a variance of 24.67% in PC1, 9.52% in PC2, and 6.68% in PC3, totaling 40.87%. In contrast, CID-44480399 (control) (**Fig 16D**) exhibited a cumulative variance of approximately 36.58%, equivalent to 21.97% from PC1, 8.76% from PC2, and 5.85% from PC3. Finally, the apoprotein (PDB ID-6GUE) (**Fig 16E**) demonstrated a variance of 13.75% in PC1, followed by 6.8% in PC2, and 6.34% in PC3, which collectively accounted for 26.89% of the total variance. The substantial conformational stability and a well-defined binding mode are suggested by the highly distinct separation of clusters along PC1 and PC2. The eigenvalue scree plot indicates that the variance contribution decreases rapidly beyond the first three PCs, indicating that the majority of conformational motions are captured within these components. In comparison to the apoprotein, all three lead compounds, CIDs-6474893, 10469828, 135438111, and the control 44480399, showed a higher proportion of variance in the first few PCs when complexed with the protein. Among them, CID-135438111 showed the least flexible and most stable binding conformation, as evidenced by a significant amount of variation. This suggests that CID-135438111 is the most stable among the analyzed complexes, followed by CID-6474893, 10469828, and the control 44480399, which exhibited relatively higher conformational fluctuations.

#### 3.7.11 DCCM analysis.

**Fig 17:**
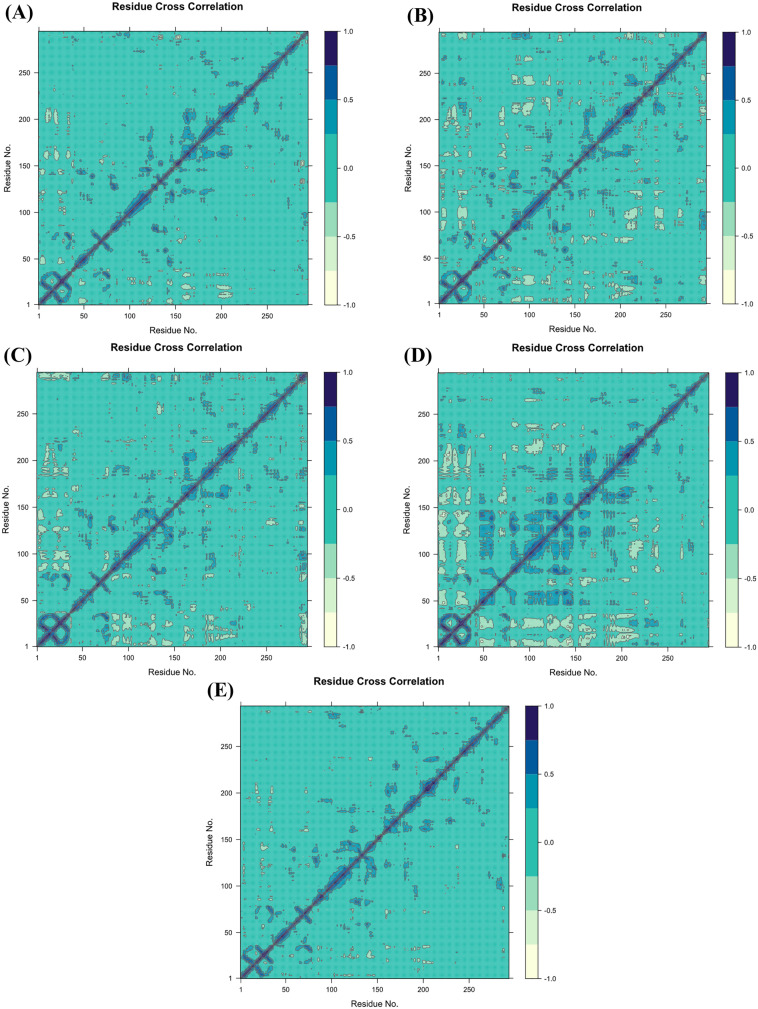
DCCM is represented by the colours blue and sea green, which signify positive and negative, respectively. Here, CIDs-6474893 (A), 10469828 (B), 135438111 (C), 44480399 (control) (D), apoprotein (PDB ID-6GUE) (E).

Protein motions occur on various time scales, including femtoseconds and seconds. DCCM is also affected by the time at which the correlation data was gathered [91]. Using inter-residue DCCM analysis, the target protein (PDB ID-6GUE), together with its docked complexes CIDs-6474893, 10469828, 135438111, control 44480399, and apoprotein (PDB ID-6GUE), were examined for correlated and anti-correlated moves. The colour blue denotes residues that are related, while the colour sea green denotes residues that are anti-correlated. The residue index maps indicated that the target protein (PDB ID-6GUE) was dispersed across populations through both positive and negative correlations. A cross-correlation map was generated by comparing the pairwise cross-correlation coefficients (**Fig 17**) between the target protein (PDB ID-6GUE) and the three lead compounds CIDs-6474893, 10469828, 135438111, and the control 44480399. The analysis revealed that CID-10469828 and 44480399 (control) had more positively correlated regions than CID-6474893 and 135438111. However, those compounds (CID-10469828 and 44480399) also exhibited a higher number of negatively correlated regions in comparison to the other two lead compounds (CIDs-6474893 and 135438111). In contrast, CID-135438111 exhibited a higher number of positively correlated regions between these two lead compounds (CID-6474893 and 135438111), while CID-6474893 had a lower number of negatively correlated regions. As a consequence, CID-6474893 demonstrated greater stability than the control (CID-44480399) and the other two lead compounds (CID-10469828 and 135438111).

## 4. Discussion

The alarmingly high incidence of colorectal cancer nowadays is quickly turning into a major issue in public health [92]. It has been difficult to create new pharmacologically effective anti-cancer treatment agents due to the lack of molecular knowledge about the oncogenic initiation and the interplay of biochemical processes and components. Recurrence is still a possibility with standard chemotherapy approaches, despite their remarkable efficacy in treating CRC [93].

Natural products are a great place to find new medicinal leads, as they include a wide variety of chemical components [94]. Novel drugs, especially those used to treat malignant illnesses, owe a great deal to their contributions [95]. Recent advancements in CRC treatment include breakthroughs in phytochemical formulations [96,97]. According to the studies, EGCG (epigallocatechin gallate) found in green tea, reduces tumor growth by inhibiting VEGFR-2 and modulating Wnt/β-catenin pathways in animal models. It also synergizes with chemotherapy to suppress cancer stem cells. Besides that, curcumin has also been shown to exhibit effects such as pro-apoptotic and anti-inflammatory via NF-κB inhibition [96]. Nanoformulations enhance their bioavailability and efficacy in CRC xenografts [97].

This study used phytochemicals from the IMPPAT database that were antimutagenic, antineoplastic, and apoptotic agonists. The effectiveness of these plant components in colon cancer therapy is still uncertain. Computational approaches were used in this investigation to find cyclin-dependent kinase (CDK) inhibitors that prevent malignant cell development and colorectal cancer. HTVS and MD simulations are effective and fast bioinformatics tools for screening multiple drug candidates [87,98]. In the present research, the goal of identifying the CDK-2 (2VSM) was to find a potential therapeutic candidate. Additionally, out of 4433 drug-like compounds, five (CIDs-135438111, 6474893, 44257567, 10469828, and 353825) exhibited increased binding affinities compared to the control (CID-44480399). These compounds were identified by protein production, ligand creation, and molecular docking studies. Furthermore, these high-affinity ligands were advanced through subsequent ADME/T profiling to assess their efficacy as potential lead compounds. Of these, three phytochemicals (CIDs-6474893, 10469828, and 135438111) demonstrated excellent properties. They did not pose any toxicological risks, and exhibited satisfactory pharmacokinetics and Lipinski criteria maintenance. Additionally, the MWs (molecular weights) of these compounds were lower compared to the control ligand. Heat molecules benefited from this since compounds with greater MWs violate the Lipinski rule [99]. The logP value is a determinant of the drug molecule’s assimilation into the organism [100]. The three compounds that were selected demonstrated logP values within the admissible range, as determined by the Lipinski criterion. The docking results were also validated using the post-docking MM-GBSA analysis. This investigation revealed that the selected three leads had more negative binding free energies than the control, which explains the greater binding affinity and the conformational stability of the leads. After that, the HOMO and LUMO energy scores were calculated for the selected compounds before the MD simulation. According to this analysis, CID-6474893 fits as the best candidate due to its highest chemical reactivity, most responsiveness to electron transfer, and least resistance to electron density change. Nevertheless, these quantum chemical descriptors obtained from this FMO analysis do not fully represent biological complexities such as solvent effects and protein flexibility. Consequently, a lower ΔE suggests the possibility of reactivity; however, it should be regarded as a supplementary factor to biological efficacy rather than a predictor of it. In this investigation, docking, MM-GBSA, ADME/T, SAR, and MD simulations were integrated with HOMO–LUMO results to facilitate a thorough assessment. In order to verify these discoveries, further experimental validation is required. For that reason, if these compounds show promise as a CRC treatment option based on MD modeling results for binding stability with the target protein, they could be worth considering. However, before starting clinical trials, it is crucial to perform in vivo and in vitro investigations. Using MD simulation, one can determine the stability and stiffness of ligand-protein complexes in an environment other than the human body. A 200 ns simulation was performed on the chosen compounds to determine the stability of their ligand-protein complexes. Using MD simulation, the following parameters of the control ligand, three lead compounds, and the protein-ligand interaction were evaluated: RMSD, RMSF, Rg, SASA, H-bonds, PCA, and DCCM. Post-simulation MM-GBSA was also run. All three lead compounds had very stable RMSDs when compared to the reference. The RMSF results showed that none of the three compounds were as unstable as the control. Based on the analysis, the average Rg of the leads was lower than the control ligand. Among them, the Rg value of CID-135438111 was the lowest, indicating that CID-135438111 can tightly pack with the protein, other than CID-44480399 (control). In the meantime, the three lead compounds had reduced SASA values compared to the control, and among the three of them, CID-10469828 displayed the lowest SASA value, indicating that it is more deeply embedded in the protein’s binding pocket in the complex system, thereby increasing its effectiveness in modulating the biological activity of the target. In the H-bond analysis, the two lead compounds (CID-6474893 and 135438111) were likely to form hydrogen bonds more strongly while binding with the target protein than the control (CID-44480399), due to their higher average H-bond values. Furthermore, in protein-ligand and ligand-protein contact analysis, CID-6474893 showed the highest stability due to its strong hydrogen bonding network, hydrophobic interactions, and engagement with key amino acid residues during the simulation. The protein-ligand binding free energy was estimated using the 200 ns dynamic simulation trajectory at both the initial and final stages (0 ns to 200 ns). According to this analysis, the control showed higher stability in the initial stage of the simulation, but it lost its stability over time, suggesting weaker binding in the long run. Comparatively, the lead compound CID-10469828 showed the best binding stability, as its binding free energy became more negative over time, suggesting enhanced stability after 200 ns. The other two lead compounds (CID-6474893 and 135438111) showed stable binding, compared to the control, but they were less favorable than CID-10469828. According to the PCA analysis, CID-135438111 showed the least flexible and most stable binding conformation, followed by CID-6474893, 10469828, and the control 44480399, which exhibited relatively higher conformational fluctuations. The DCCM study demonstrated that CID-6474893 had a higher level of stability than the control (CID-44480399), as evidenced by the fact that it had a lower number of negatively correlated regions. Despite the fact that CID-44480399 (control) had a greater number of positively correlated regions than the three lead compounds, it also had a greater number of negatively correlated regions, which exceeded the criteria of having a higher number of positively correlated regions. Thus, it can be concluded that the control did not perform well in any of the analyses in comparison to the three lead compounds (CIDs-6474893, 10469828, and 135438111), and CID-6474893 performed best among them. Here, CID-6474893 ((1E,4E)-1,5-bis(4-hydroxy-3-methoxyphenyl) penta-1,4-dien-3-one) and CID-10469828 ((1E,4E)-1-(4-Hydroxy-3-methoxyphenyl)-5-(4-hydroxyphenyl)-1,4-pentadien-3-one) were retrieved from *Curcuma longa*, whether CID-135438111 was from *Peganum harmala*. As these lead compounds have not been previously reported, their identification in this study marks the first instance of their recruitment, suggesting their potential for further research in the development of CRC treatment.

In cheminformatics-based drug design, we used a computer-aided algorithm to simulate the biological system. But in reality, the biological system is quite complex, and the dynamic nature of protein flexibility is unstable [101]. An *in silico* model can’t count all off-target effects as it doesn’t deal with the whole cellular system. In a biological system, the binding of a ligand to a receptor initiates a downstream signaling cascade, whereas *in silico* analysis focuses solely on its targeted effect or process. ADME/T analysis is a prediction-based method to calculate all its possible outcomes. But in reality, it doesn’t reflect a drug’s pharmacokinetic properties. It can only give a primary guess. So, in vitro and in vivo validation is a crying need for discovering a new therapeutic drug. An *in vitro* model works like a bridge between computational predictions and real-world biology, and *in vivo* analysis shows the overall effect of a drug in a living organism, where all physiological processes of a biological system are interconnected [102]. Our *in silico* drug design method against CRC treatment was completely a computer-based analysis, which is also known as a pre-clinical study. As a consequence of our laboratory support and funding, we were unable to expand our research findings. Consequently, individuals who are involved in the treatment of this disease may utilize this outcome as a basis for their *in vivo*/*in vitro* validation.

## 5. Conclusion

Cheminformatics-based drug design was employed to simulate the druggability of phytochemicals against colorectal cancer. Phytochemicals that target the enzyme CDK-2 may prevent the progression of cancer through halting the spread of tumors and killing off cancer-causing stem cells by influencing their activity. The main objective of this study is to identify potential lead chemicals that can inhibit cancer progression. Because of this, the objective of the investigation is to determine whether the leads exhibit superior efficacy in preventing the progression of cancer compared to a commercially available drug. In this investigation, molecular docking, ADME/T, protein-ligand interaction, MM-GBSA, SAR, FMO, and MD simulations identified three compounds (CIDs-6474893, 10469828, and 135438111) that have the potential to inhibit the activity of CDK-2, thereby facilitating the development of an anticancer drug. However, in order to verify the compounds’ efficacy against cancer, *in vivo* and *in vitro* studies are necessary.

## Supporting information

S1 File Anticancer plant list. (ZIP)
