# Supplementary material for: Cheminformatics-based screening and evaluation of phytochemicals as CDK2 inhibitors in colorectal cancer therapy
Source: PLoS One. 2025 Sep 3;20(9):e0331438. doi: 10.1371/journal.pone.0331438 (PMC12407419; doi:10.1371/journal.pone.0331438)
Supplement: S1 File — (ZIP) [file pone.0331438.s001.zip › S1_Anticancer plant list.docx]

| **Scientific Name of the Plant** | **Local Name in Bangladesh** | **The Activity of the Plants for Different Cancers** | **References** |
| --- | --- | --- | --- |
| *Acorus calamus* | Bach/Bacha | Skin cancer | [1], [2] |
| *Aegle marmelos* | Bel | Human cervical cancer cells (HeLa) | [3], [4] |
| *Agrimonia Pilosa* | Hairy Agrimony | Colon cancer | [5], [6] |
| *Asclepias curassavicap* | Moricha | Anticancer  activities | [7] |
| *Berberis vulgaris* | Daruchini | Breast cancer. | [8], [9] |
| *Ceiba pentandra* | Shimul | Anticancer  activities | [10] |
| *Citrus limon* | Lemon | Human cervical cancer cell line | [11], [12] |
| *Coccinia grandis* | Korola | Anticancer  activities | [13] |
| *Curcuma longa* | Holud | Lung cancer | [14], [15] |
| *Glycyrrhiza glabra* | Mitha Madhurika | Breast cancer | [16], [17] |
| *Mallotus philippensis* | Kakamachi | Anticancer  activities | [18] |
| *Mentha arvensis* | Pudina | Lung cancer | [19], [20] |
| *Moringa oleifera* | Shojne | Breast cancer | [21], [22] |
| *Panax ginseng* | Ashwagandha | Breast cancer | [23], [24] |
| *Peganum harmala* | Haritaki | Anticancer  activities | [25] |
| *Pongamia pinnata* | Kachi Nim | MCF-7 breast cancer cells | [26], [27] |
| *Ruta graveolens* | Sadapata | Colon cancer | [28], [29] |
| *Trapa natans* | Singra | A431 human skin cancer | [30] |
| *Viscum album* | Banda Pata/ Banda shak | Melanoma murine cancer cells | [31], [32] |
| Solanum nigrum | Makarcha | Breast cancer | [33], [34] |

**Table**: Medicinal Plants with Reported Anticancer Activities Against Various Cancer Types.

**Reference**

[1] R. Vakayil *et al.*, “Acorus calamus-zinc oxide nanoparticle coated cotton fabrics shows antimicrobial and cytotoxic activities against skin cancer cells,” *Process Biochem.*, vol. 111, pp. 1–8, Dec. 2021, doi: 10.1016/J.PROCBIO.2021.08.024.

[2] B. K. Das, A. V. Swamy, B. C. Koti, and P. C. Gadad, “Experimental evidence for use of Acorus calamus (asarone)for cancer chemoprevention,” *Heliyon*, vol. 5, no. 5, May 2019, doi: 10.1016/j.heliyon.2019.e01585.

[3] H. Bobade, S. Sharma, and A. Singh, “Indian Bael,” *Antioxidants Fruits Prop. Heal. Benefits*, pp. 135–161, 2020, doi: 10.1007/978-981-15-7285-2_8.

[4] D. T. Sukumar, G. Gunasangkaran, V. A. Arumugam, and S. Muthukrishnan, “Effects of biogenic synthesis of chitosan entrapped silver nanoparticle from Aegle marmelos on human cervical cancer cells (HeLa),” *J. Drug Deliv. Sci. Technol.*, vol. 70, p. 103189, Apr. 2022, doi: 10.1016/J.JDDST.2022.103189.

[5] N. T. Trinh, T. M. N. Nguyen, J. I. Yook, S. G. Ahn, and S. A. Kim, “Quercetin and Quercitrin from Agrimonia pilosa Ledeb Inhibit the Migration and Invasion of Colon Cancer Cells through the JNK Signaling Pathway,” *Pharmaceuticals*, vol. 15, no. 3, p. 364, Mar. 2022, doi: 10.3390/PH15030364.

[6] T. Y. Kim *et al.*, “Proteomics Analysis of Antitumor Activity of Agrimonia pilosa Ledeb. in Human Oral Squamous Cell Carcinoma Cells,” *Curr. Issues Mol. Biol. 2022, Vol. 44, Pages 3324-3334*, vol. 44, no. 8, pp. 3324–3334, Jul. 2022, doi: 10.3390/CIMB44080229.

[7] A. E. Al-Snafi, “The medical Importance of Cicer arietinum-A review,” *IOSR J. Pharm. www.iosrphr.org*, vol. 6, no. 3, pp. 29–40, 2016.

[8] E. Ghafourian *et al.*, “Ethanolic Extract of Berberis Vulgaris Fruits Inhibits the Proliferation of MCF-7 Breast Cancer Cell Line Through Induction of Apoptosis,” *Infect. Disord. - Drug Targets*, vol. 17, no. 3, May 2017, doi: 10.2174/1871526517666170531113759.

[9] M. Gulfishan, M. Afzal, I. Kazmi, A. M. Quazi, T. A. Bhat, and A. Jahan, “Mechanism of action of anticancer herbal medicines,” *Anticancer Plants Mech. Mol. Interact.*, vol. 4, pp. 337–360, Jul. 2018, doi: 10.1007/978-981-10-8417-1_14/COVER.

[10] R. Kumar, N. Kumar, G. V. Ramalingayya, M. M. Setty, and K. S. R. Pai, “Evaluation of Ceiba pentandra (L.) Gaertner bark extracts for in vitro cytotoxicity on cancer cells and in vivo antitumor activity in solid and liquid tumor models,” *Cytotechnology*, vol. 68, no. 5, pp. 1909–1923, Oct. 2016, doi: 10.1007/S10616-016-0002-2/METRICS.

[11] H. I. Al Othman *et al.*, “Phytochemical Composition, Antioxidant and Antiproliferative Activities of Citrus hystrix, Citrus limon, Citrus pyriformis, and Citrus microcarpa Leaf Essential Oils against Human Cervical Cancer Cell Line,” *Plants*, vol. 12, no. 1, p. 134, Jan. 2023, doi: 10.3390/PLANTS12010134/S1.

[12] M. Osanloo, A. Ghanbariasad, and A. Taghinezhad, “Antioxidant and Anticancer Activities of Anethum graveolens L., Citrus limon (L.) Osbeck and Zingiber officinale Roscoe Essential Oils,” *Tradit. Integr. Med.*, vol. 6, no. 4, pp. 333–347, Sep. 2021, doi: 10.18502/TIM.V6I4.8266.

[13] “(6) In vivo and in vitro anticancer activity of Coccinia grandis (L.) Voigt (Family: Cucurbitaceae) on Swiss albino mice | Request PDF.”

[14] P. Maheswari, S. Harish, M. Navaneethan, C. Muthamizhchelvan, S. Ponnusamy, and Y. Hayakawa, “Bio-modified TiO2 nanoparticles with Withania somnifera, Eclipta prostrata and Glycyrrhiza glabra for anticancer and antibacterial applications,” *Mater. Sci. Eng. C*, vol. 108, p. 110457, Mar. 2020, doi: 10.1016/J.MSEC.2019.110457.

[15] W. Kukula-Koch *et al.*, “Superior anticancer activity is demonstrated by total extract of Curcuma longa L. as opposed to individual curcuminoids separated by centrifugal partition chromatography,” *Phyther. Res.*, vol. 32, no. 5, pp. 933–942, May 2018, doi: 10.1002/PTR.6035.

[16] S. Dong, A. Inoue, Y. Zhu, M. Tanji, and R. Kiyama, “Activation of rapid signaling pathways and the subsequent transcriptional regulation for the proliferation of breast cancer MCF-7 cells by the treatment with an extract of Glycyrrhiza glabra root,” *Food Chem. Toxicol.*, vol. 45, no. 12, pp. 2470–2478, Dec. 2007, doi: 10.1016/J.FCT.2007.05.031.

[17] P. Mohammad, Z. Nosratollah, R. Mohammad, A. Abbas, and R. Javad, “The inhibitory effect of Curcuma longa extract on telomerase activity in A549 lung cancer cell line,” *African J. Biotechnol.* , vol. 9, no. 6, pp. 912–919, Feb. 2010, doi: 10.5897/AJB09.904.

[18] V. Sharma, “A polyphenolic compound rottlerin demonstrates significant in vitro cytotoxicity against human cancer cell lines: isolation and characterization from the fruits of Mallotus philippinensis,” *J. Plant Biochem. Biotechnol.*, vol. 20, no. 2, pp. 190–195, 2011, doi: 10.1007/s13562-011-0045-6.

[19] P. Sakthidhasan, P. S. Kumar, and M. B. G. Viswanathan, “ Apoptotic and Antiproliferative Potential of GAPDH from Mallotus philippensis Seed on Human Lung Carcinoma: In Vitro and In Vivo Approach ,” *Protein Pept. Lett.*, vol. 29, no. 4, pp. 340–349, Mar. 2022, doi: 10.2174/0929866529666220302104935.

[20] A. I. Hussain, F. Anwar, P. S. Nigam, M. Ashraf, and A. H. Gilani, “Seasonal variation in content, chemical composition and antimicrobial and cytotoxic activities of essential oils from four mentha species,” *J. Sci. Food Agric.*, vol. 90, no. 11, pp. 1827–1836, Aug. 2010, doi: 10.1002/jsfa.4021.

[21] I. A. Adebayo, H. Arsad, and M. R. Samian, “ANTIPROLIFERATIVE EFFECT ON BREAST CANCER (MCF7) OF MORINGA OLEIFERA SEED EXTRACTS,” *African J. Tradit. Complement. Altern. Med. AJTCAM*, vol. 14, no. 2, pp. 282–287, 2017, doi: 10.21010/AJTCAM.V14I2.30.

[22] D. Barhoi, P. Upadhaya, S. N. Barbhuiya, A. Giri, and S. Giri, “Aqueous Extract of Moringa oleifera Exhibit Potential Anticancer Activity and can be Used as a Possible Cancer Therapeutic Agent: A Study Involving In Vitro and In Vivo Approach,” *J. Am. Coll. Nutr.*, vol. 40, no. 1, pp. 70–85, 2021, doi: 10.1080/07315724.2020.1735572.

[23] S. J. Kim and A. K. Kim, “Anti-breast cancer activity of Fine Black ginseng (Panax ginseng Meyer) and ginsenoside Rg5,” *J. Ginseng Res.*, vol. 39, no. 2, pp. 125–134, Apr. 2015, doi: 10.1016/J.JGR.2014.09.003.

[24] T. V. M. Sreekanth *et al.*, “Ultra-sonication-assisted silver nanoparticles using Panax ginseng root extract and their anti-cancer and antiviral activities,” *J. Photochem. Photobiol. B Biol.*, vol. 188, pp. 6–11, Nov. 2018, doi: 10.1016/J.JPHOTOBIOL.2018.08.013.

[25] A. Jalali, F. Dabaghian, and M. M. Zarshenas, “Alkaloids of Peganum harmala: Anticancer Biomarkers with Promising Outcomes,” *Curr. Pharm. Des.*, vol. 27, no. 2, pp. 185–196, Nov. 2020, doi: 10.2174/1381612826666201125103941.

[26] B. Malaikozhundan *et al.*, “Biological therapeutics of Pongamia pinnata coated zinc oxide nanoparticles against clinically important pathogenic bacteria, fungi and MCF-7 breast cancer cells,” *Microb. Pathog.*, vol. 104, pp. 268–277, Mar. 2017, doi: 10.1016/J.MICPATH.2017.01.029.

[27] G. Chen *et al.*, “A natural chalcone induces apoptosis in lung cancer cells: 3D-QSAR, docking and an in vivo/vitro assay,” *Sci. Reports 2017 71*, vol. 7, no. 1, pp. 1–10, Sep. 2017, doi: 10.1038/s41598-017-11369-9.

[28] Y. Tsai, “Social security income and the utilization of home care: Evidence from the social security notch,” *J. Health Econ.*, vol. 43, pp. 45–55, Sep. 2015, doi: 10.1016/J.JHEALECO.2014.10.001.

[29] P. Varamini, M. Soltani, and A. Ghaderi, “Cell cycle analysis and cytotoxic potential of Ruta graveolens against human tumor cell lines,” *Neoplasma*, vol. 56, no. 6, pp. 490–493, 2009, doi: 10.4149/NEO_2009_06_490.

[30] M. M. Saber, S. B. Mirtajani, and K. Karimzadeh, “Green synthesis of silver nanoparticles using Trapa natans extract and their anticancer activity against A431 human skin cancer cells,” *J. Drug Deliv. Sci. Technol.*, vol. 47, pp. 375–379, Oct. 2018, doi: 10.1016/J.JDDST.2018.08.004.

[31] T. Ćebović, S. Spasić, and M. Popović, “Cytotoxic effects of the Viscum album L. extract on ehrlich tumour cells in vivo,” *Phyther. Res.*, vol. 22, no. 8, pp. 1097–1103, Aug. 2008, doi: 10.1002/ptr.2464.

[32] M. N. de O. Melo *et al.*, “Phenolic compounds from Viscum album tinctures enhanced antitumor activity in melanoma murine cancer cells,” *Saudi Pharm. J.*, vol. 26, no. 3, pp. 311–322, Mar. 2018, doi: 10.1016/J.JSPS.2018.01.011.

[33] Y. J. Lai *et al.*, “Anti-Cancer Activity of Solanum nigrum (AESN) through Suppression of Mitochondrial Function and Epithelial-Mesenchymal Transition (EMT) in Breast Cancer Cells,” *Mol. 2016, Vol. 21, Page 553*, vol. 21, no. 5, p. 553, Apr. 2016, doi: 10.3390/MOLECULES21050553.

[34] F. Shi *et al.*, “Preparative isolation and purification of steroidal glycoalkaloid from the ripe berries of Solanum nigrum L. by preparative HPLC–MS and UHPLC–TOF-MS/MS and its anti-non-small cell lung tumors effects in vitro and in vivo,” *J. Sep. Sci.*, vol. 42, no. 15, pp. 2471–2481, Aug. 2019, doi: 10.1002/JSSC.201801165.
