# Supplementary material for: Cheminformatics-based screening and evaluation of phytochemicals as CDK2 inhibitors in colorectal cancer therapy
Source: PLoS One. 2025 Sep 3;20(9):e0331438. doi: 10.1371/journal.pone.0331438 (PMC12407419; doi:10.1371/journal.pone.0331438)
Supplement: S1 File — (ZIP) [file pone.0331438.s001.zip › S2_ADMET.docx]

**Table 1:** Pharmacokinetic properties of the top five phytochemical compounds CID-135438111, 6474893, 44257567, and 353825**,** containing higher negative docking scores, compared to the control CID-44480399.

| **Properties** | | **CID-135438111 (IMPHY008184)** | **CID-6474893 (IMPHY007097)** | **CID-44257567 (IMPHY013402)** | **CID-10469828 (IMPHY014179)** | **CID-353825 (IMPHY011367)** | **CID- 44480399 (Control)** |
| --- | --- | --- | --- | --- | --- | --- | --- |
| Docking score | | -9.271 | -9.255 | -9.255 | -9.218 | -9.176 | -8.037 |
| Physiological Properties | Formula | C11H12N2O2 | C19H18O5 | C18H12O4 | C18H16O4 | C21H19N3O5 | C17H13NO3 |
|  | MW (g/mol) | 204.23 | 326.34 | 292.29 | 296.32 | 393.39 | 279.29 |
|  | Heavy atoms | 15 | 24 | 22 | 22 | 29 | 22 |
|  | Arom. Heavy | 10 | 12 | 19 | 12 | 19 | 16 |
|  | Rotatable bonds | 3 | 6 | 2 | 5 | 6 | 1 |
|  | H-bond acceptor | 3 | 5 | 4 | 4 | 7 | 3 |
|  | H-bond donors | 2 | 2 | 0 | 2 | 1 | 0 |
|  | Molar Refractivity | 58.11 | 92.99 | 84.18 | 86.5 | 106.77 | 80.97 |
| Lipophilicity | Consensus log *P* | 1.42 | 3.05 | 3.4 | 3.03 | 3.2 | 2.88 |
| Water solubility | Log S (ESOL) | -1.89 | -3.95 | -4.4 | -3.93 | -4.53 | -4.05 |
|  | Solubility | 2.61e+00 mg/ml ; 1.28e-02 mol/l | 7.77e-03 mg/ml ; 2.38e-05 mol/l | 1.18e-02 mg/ml ; 4.02e-05 mol/l | 3.48e-02 mg/ml ; 1.17e-04 mol/l | 1.16e-02 mg/ml ; 2.96e-05 mol/l | 2.47e-02 mg/ml ; 8.83e-05 mol/l |
|  | Class | Very soluble | Moderately soluble | Moderately soluble | Soluble | Moderately soluble | Moderately soluble |
| Pharmacokinetics | GI absorption | High | High | High | High | High | High |
| Drug Likeness | Lipsinki, Violation | Yes; 0 violation | Yes; 0 violation | Yes; 0 violation | Yes; 0 violation | Yes; 0 violation | Yes; 0 violation |
|  | Bioavailabity | 0.55 | 0.55 | 0.55 | 0.55 | 0.55 | 0.55 |
| Medical Chemistry | Synthetic accesability | 2.03 | 2.76 | 3.02 | 2.61 | 3.34 | 2.55 |

**Table 2:** Toxicity properties of the top five phytochemical compounds CID-135438111, 6474893, 44257567, 353825, and control 44480399.

| **Classification** | **Properties** | | **CID-135438111 (IMPHY008184)** | **CID-6474893 (IMPHY007097)** | **CID-44257567 (IMPHY013402)** | **CID-10469828 (IMPHY014179)** | **CID-353825 (IMPHY011367)** | **CID- 44480399 (Control)** |
| --- | --- | --- | --- | --- | --- | --- | --- | --- |
|  | LD 50 (mg/kg) | | 1100 | 4000 | 4000 | 4000 | 400 | 1000 |
|  | Toxicity Class | | 4 | 5 | 5 | 5 | 4 | 4 |
| Organ Toxicity | Hepatotoxicity | Prediction | Inactive | Inactive | Inactive | Inactive | Inactive | Inactive |
|  |  | Probability | 0.64 | 0.59 | 0.75 | 0.58 | 0.65 | 0.61 |
|  | Neurotoxicity | Prediction | Active | Inactive | Inactive | Inactive | Active | Active |
|  |  | Probability | 0.59 | 0.72 | 0.81 | 0.76 | 0.66 | 0.52 |
|  | Nephrotoxicity | Prediction | Inactive | Active | Inactive | Active | Active | Inactive |
|  |  | Probability | 0.52 | 0.54 | 0.54 | 0.54 | 0.55 | 0.63 |
|  | Respiratory Toxicity | Prediction | Active | Inactive | Active | Inactive | Active | Inactive |
|  |  | Probability | 0.74 | 0.68 | 0.6 | 0.66 | 0.66 | 0.52 |
|  | Cardiotoxicity | Prediction | Inactive | Inactive | Inactive | Inactive | Inactive | Inactive |
|  |  | Probability | 0.74 | 0.76 | 0.68 | 0.66 | 0.72 | 0.85 |
| Toxicity End Products | Carcinogenicity | Prediction | Inactive | Inactive | Active | Inactive | Active | Active |
|  |  | Probability | 0.63 | 0.69 | 0.75 | 0.66 | 0.54 | 0.54 |
|  | Immunotoxicity | Prediction | Inactive | Active | Inactive | Active | Inactive | Active |
|  |  | Probability | 0.98 | 0.79 | 0.89 | 0.85 | 0.57 | 0.99 |
|  | Mutagenicity | Prediction | Inactive | Inactive | Active | Inactive | Active | Active |
|  |  | Probability | 0.62 | 0.76 | 0.55 | 0.7 | 0.65 | 0.7 |
|  | Cytotoxicity | Prediction | Inactive | Inactive | Inactive | Inactive | Inactive | Inactive |
|  |  | Probability | 0.81 | 0.96 | 0.74 | 0.84 | 0.5 | 0.62 |
|  | BBB barrier | Prediction | Active | Active | Active | Active | Active | Active |
|  |  | Probability | 0.78 | 0.62 | 0.69 | 0.63 | 0.84 | 0.79 |
|  | Ecotoxicity | Prediction | Inactive | Inactive | Active | Inactive | Active | Active |
|  |  | Probability | 0.66 | 0.65 | 0.66 | 0.71 | 0.58 | 0.67 |
|  | Clinical Toxicity | Prediction | Active | Inactive | Inactive | Inactive | Active | Active |
|  |  | Probability | 0.7 | 0.53 | 0.51 | 0.52 | 0.58 | 0.57 |
|  | Nutrotional Toxicity | Prediction | Inactive | Inactive | Active | Inactive | Inactive | Inactive |
|  |  | Probability | 0.68 | 0.81 | 0.56 | 0.82 | 0.64 | 0.52 |
| Metabolism | Cytochrome CYP1A2 | Prediction | Inactive | Active | Active | Active | Active | Active |
|  |  | Probability | 0.72 | 0.54 | 0.98 | 0.67 | 0.61 | 0.7 |
|  | Cytochrome CYP2C19 | Prediction | Inactive | Active | Active | Active | Active | Inactive |
|  |  | Probability | 0.76 | 0.79 | 0.91 | 0.81 | 0.71 | 0.75 |
|  | Cytochrome CYP2C9 | Prediction | Inactive | Active | Active | Active | Inactive | Inactive |
|  |  | Probability | 0.62 | 0.81 | 0.96 | 0.77 | 0.82 | 0.5 |
|  | Cytochrome CYP2D6 | Prediction | Inactive | Inactive | Active | Inactive | Active | Inactive |
|  |  | Probability | 0.65 | 0.69 | 0.78 | 0.69 | 0.86 | 0.76 |
|  | Cytochrome CYP3A4 | Prediction | Inactive | Active | Active | Inactive | Active | Active |
|  |  | Probability | 0.77 | 0.8 | 0.96 | 0.59 | 0.95 | 0.52 |
|  | Cytochrome CYP2E1 | Prediction | Inactive | Inactive | Inactive | Inactive | Inactive | Inactive |
|  |  | Probability | 0.99 | 1 | 0.95 | 1 | 0.99 | 1 |
