# Supplementary material for: Cheminformatics-based screening and evaluation of phytochemicals as CDK2 inhibitors in colorectal cancer therapy
Source: PLoS One. 2025 Sep 3;20(9):e0331438. doi: 10.1371/journal.pone.0331438 (PMC12407419; doi:10.1371/journal.pone.0331438)
Supplement: S1 File — (ZIP) [file pone.0331438.s001.zip › S3_Post-docking MM-GBSA.docx]

**Table:** Post-docking MM-GBSA analysis results

| **CID** | **6474893** | **10469828** | **135438111** | **44480399** |
| --- | --- | --- | --- | --- |
| ΔG Bind | -59.79 | -55.64 | -46.99 | -40.39 |
| ΔG Bind Coulomb | -24.65 | -20.19 | -21.61 | -16.43 |
| ΔG Bind Covalent | 6.07 | 4.98 | 1.06 | 16.98 |
| ΔG Bind Hbond | -3.20 | -2.87 | -1.57 | -1.58 |
| ΔG Bind Lipo | -27.70 | -27.11 | -9.58 | -15.44 |
| ΔG Bind Packing | -1.39 | -1.37 | -0.76 | -1.33 |
| ΔG Bind Solv GB | 35.45 | 32.24 | 15.91 | 26.87 |
| ΔBind vdW | -44.38 | -41.32 | -30.43 | -49.45 |
